# Risk factors associated with the occurrence of anthrax outbreaks in livestock in the country of Georgia: A case-control investigation 2013-2015

**DOI:** 10.1371/journal.pone.0215228

**Published:** 2019-05-02

**Authors:** Sangeeta Rao, Rita Traxler, Tsira Napetavaridze, Zviad Asanishvili, Ketevan Rukhadze, Giorgi Maghlakelidze, Marika Geleishvili, Mariam Broladze, Maka Kokhreidze, Debby Reynolds, Sean Shadomy, Mo Salman

**Affiliations:** 1 Department of Clinical Sciences, Colorado State University, Fort Collins, Colorado, United States of America; 2 Centers for Disease Control and Prevention (CDC), Division of High-Consequence Pathogens and Pathology, National Center for Emerging and Zoonotic Infectious Diseases (NCEZID), Atlanta, Georgia, United States of America; 3 National Food Agency (NFA) of Ministry of Environmental Protection and Agriculture of Georgia (MEPA), Tbilisi, Georgia; 4 Department of Rural Development and Vocational Education (DRDVE) of Georgian Institute of Public Affairs (GIPA), Tbilisi, Georgia; 5 National Center for Disease Control and Prevention, Tbilisi, Georgia; 6 Laboratory of the Ministry of Agriculture (LMA), Tbilisi, Georgia; 7 CDC One Health Office, NCEZID, Atlanta, Georgia, United States of America; National Veterinary School of Toulouse, FRANCE

## Abstract

**Introduction:**

Anthrax is considered endemic in livestock in Georgia. In 2007, the annual vaccination became the responsibility of livestock owners, while contracting of private veterinarians was not officially required. Six years later, due to increase in human outbreaks associated with livestock handling, there is a need to find out the risk factors of livestock anthrax in Georgia.

**Objective:**

To identify exposures and risk factors associated with livestock anthrax.

**Methods:**

A matched case-control study design was used to recruit the owners of individual livestock anthrax cases that occurred between June 2013 and May 2015, and owners of unaffected livestock from within (“village control”) and outside the village (“area control”).

We collected data about the case and control livestock animals’ exposure and risk factors within the one-month prior to the disease onset of the case livestock (or matched case for the controls). We used logistic regression analysis (univariate and multivariable) to calculate the odds ratios of exposures and risk factors.

**Results:**

During the study period, 36 anthrax cases met the case definition and were enrolled in the study; 67 matched village control livestock and 71 matched area control livestock were also enrolled.

The findings from multivariable logistic regression analysis demonstrate that vaccination within the last two years significantly reduced the odds of anthrax in cattle (OR = 0.014; 95% Confidence interval = <0.001, 0.99). The other factors that were significantly protective against anthrax were ‘animals being in covered fence area/barn’ (OR = 0.065; p-value = 0.036), and ‘female animal being pregnant or milking compared to heifer’ (OR = 0.006; p-value = 0.037).

**Conclusions:**

The information obtained from this study has involved and been presented to decision makers, used to build technical capacity of veterinary staff, and to foster a One Health approach to the control of zoonotic diseases which will optimize prevention and control strategies. Georgia has embedded the knowledge and specific evidence that vaccination is a highly protective measure to prevent anthrax deaths among livestock, to which primary emphasis of the anthrax control program will be given. Education of livestock keepers in Georgia is an overriding priority.

## Introduction

Zoonotic potential of the disease anthrax is well known worldwide. Anthrax is a bacterial disease affecting humans and other mammals and caused by the gram-positive, spore-forming, rod-shaped bacterium *Bacillus anthracis* [1]. Grazing livestock are thought to become infected when they ingest *B*. *anthracis* spores on vegetation or in soil consumed with vegetation or its roots, or from water sources where the spores may become concentrated. The soil and water are contaminated from carcasses or burial sites of previously infected livestock [2, 3, 4]. Livestock infections can also occur when grass or hay cut from contaminated fields, or livestock feed or supplements such as mineral supplements manufactured with bone meal from infected carcasses, are fed to livestock.

Human infection primarily results from contact with or handling of infected livestock, or the carcasses of infected livestock [5], or from contact with infected livestock products such as meat, hides, or bone or through consumption of raw and/or undercooked infected meat or blood, or contact with infected animal products (hides, wool, hooves, etc). The incidence of anthrax varies geographically, but the disease occurs globally. Georgia is a country in the Caucasus region in the junction of Western Asia and Eastern Europe, regions that are endemic for anthrax disease [6].

Outbreaks in livestock have been found to be associated with low-lying areas [4, 7] where the soil has high moisture and organic content, or with soils with alkaline pH or high calcium ion concentrations [8, 9]. Anthrax spores that are present in carcass remains and old burial sites of infected livestock are still a potential danger in most regions of the world, especially should anything happen to upset recognized practices of hygiene and control [10]. Biting flies may act as mechanical vectors, and contribute to disease spread, and carrion-eating flies may contaminate vegetation after feeding on the infected carcasses [10, 11].

Generally, there are many factors that have been documented in the past for human anthrax cases that include occupational exposure to infected livestock, their carcasses, or to products from infected livestock (e.g. meat, wool, hides) [12]. There are many studies and investigations that have identified risk factors for human anthrax [5, 6, 13, 14, 15, 16, 17]. However, there is a lack of studies that looked at risk factors for anthrax in livestock; more specifically no study was conducted in Georgia.

Anthrax is considered endemic in Georgia [6, 17, 18,]. From 2007 to 2013, there were 92 officially reported cases of anthrax in cattle (83), sheep (7) horse (1) and pig (1) in Georgia, and a total of 358 human cases. In 2012, there was a fivefold increase in human cases compared to 2010 [6]. An epidemiologic investigation in 2012 of human anthrax cases demonstrated a strong association with disposal of dead livestock, participation in livestock slaughter, caring for sick livestock, handling livestock products, or owning or working with livestock within the month prior to onset of the human case [6].

A 1995 law required the prevention of epizootic diseases in Georgia, which included mandatory livestock anthrax vaccinations; however, in 2007, responsibility shifted from state-funded annual vaccination campaigns to livestock owners contracting private veterinarians to provide anthrax vaccinations [6]. The spike in livestock anthrax cases may be due to this change with decreased vaccination coverage due to cost implication to the livestock owner. Between the years 2000 and 2012, the National Food Agency (NFA) reported 120 livestock anthrax cases; the number of reported livestock cases increased threefold between 2010 and 2012 [6]. It is, however, assumed that livestock cases are underreported, and livestock cases are usually detected retrospectively after a human case has been reported [1]. Human anthrax was highly prevalent in Kvemo Kartli and Kakheti regions, and these regions comprised 40% of Georgia’s susceptible livestock [6].

As a result of the outbreak investigation findings in 2012, anthrax control is now an ongoing part of the National Animal Health Program in Georgia with regular prophylactic vaccination campaigns of livestock provided by the NFA from 2013 (Lasha Avaliani, Head of Veterinary Department NFA at the time, personal communication). There was, however, a knowledge gap regarding the specific and most important exposure and risk factors associated with the occurrence and the spread of anthrax in livestock in Georgia. There remained a need to mirror the epidemiological investigation of human anthrax with one focused on livestock. To address these gaps, we conducted a case control investigation to compare the exposures and other factors of livestock anthrax cases and living control livestock over two years.

## Materials and methods

### Study design

A matched case-control study design was used to recruit the owners of individual livestock anthrax cases that occurred between June 2013 and May 2015, and owners of unaffected livestock to serve as controls. The unit of the study was individual animal, where case and control animals were matched on the same species. There was no matching done by age or sex, as these both may be potential risk factors and may also influence or confound other factors. The study survey addressed the condition, care and other elements of management of the particular case or control animal during the period of ‘30 days prior to onset of disease’ in the case animal for each of the case-control pairings; if the date of onset is not available, the reported date of death was used based on the short course of disease in livestock.

#### Study sample and definitions

The case definition used for anthrax confirmation was: “A Suspect or Probable anthrax case and culture positive for *B*. *anthracis* or PCR detection of *B*. *anthracis*”. [1].

A Suspect or Probable Anthrax Case was an animal of one of the target livestock species (cattle; sheep; goats; pigs; and horses or donkeys) meeting the following criteria established by NFA:

- Suspect Anthrax Case—Sudden unexplained death in a target species, especially in a known anthrax infected area, or where there is an epidemiological link to a herd with a history of anthrax. The carcass may have poorly clotted blood exuding from natural orifices, or animal may exhibit a large spleen on postmortem.
- Probable Anthrax Case—any of the following may be classified as probable cases:
  - A target species exhibiting any of high fever, malaise, dyspnoea, dysentery or mucosal congestion followed by terminal convulsions and death;
  - The carcass of a target species exhibiting incomplete rigor mortis and un-clotted blood exuding from the anus, vulva, nostrils and/or mouth;
  - A carcass that meets the suspect case definition with demonstration of encapsulated *B*. *anthracis* in smears of blood or tissues stained with polychrome methylene blue (M’Fadyean) stain or Giemsa stain.
- Confirmed Anthrax Case—A Suspect or Probable Anthrax Case which meets the following diagnostic laboratory criteria:
  - Isolation and identification of *B*. *anthracis* by Laboratory of the Ministry of Agriculture (LMA)or other relevant laboratory;
  - PCR detection of *B*. *anthracis* virulence factor nucleic acid by LMA or other relevant laboratory test.

A “Case Animal” based on the “Confirmed Anthrax Case” definition, was identified and considered for inclusion in this investigation based on:

1. Livestock that were identified through reporting information recorded in the Electronic Integrated Disease Surveillance System (EIDSS) [19], which stores case information on reportable human and veterinary diseases (National Center for Disease Control and Public Health, 2018), and at NFA from the original livestock anthrax investigation;
2. Livestock that were identified as ‘Confirmed Anthrax Cases’ in the NFA surveillance records and investigation reports;
3. Livestock that were identified as ‘Confirmed Anthrax Cases’ which were found through follow-up investigation of human anthrax cases that were reported to National Center for Disease Control and Prevention (NCDC);
4. The livestock had a date of onset of anthrax illness between 1 June 2013 and 31 May 2015; if the date of onset was not available, the reported date of death was used based on the short course of disease in livestock;
5. Case occurred in one of five regions most heavily affected with livestock anthrax since 2007 (Samtskhe-Javakheti, Kakheti, Kvemo Kartli, Samegrelo and Imereti).

For this study, the term “Animal Management Unit” (AMU) was used to define a group of livestock from a single settlement or village that were gathered, managed and fed, and pastured together as a single group. There may be multiple owners of livestock within a single AMU. In addition, there may be different AMUs from within the same village or settlement which are managed separately.

#### Control selection, inclusion and exclusion criteria

Once a case was enrolled in the study and the survey was completed, the surveyor or study staff member randomly selected two “Village” controls. Two-stage simple random sampling was used to select two separate households within the same settlement using a randomly selected number of buildings away from the case owner’s home; once the house was selected, an animal was randomly selected from amongst those owned by the household. The selected household must have had at least one animal of the same species as the case; the selected animal must have been greater than 3 months of age and present in the case’s village during the same one-month period prior to the case’s disease onset or date of death. If no one was present in the selected house, the house was vacant, or the household members declined to participate, the study staff moved to the next household until a household was selected and household members consented to participate.

Two “Geographic Area” controls for each Case Animal were selected using multi-stage sampling with simple random sampling at each stage. First, we randomly selected two bearings and azimuths on a compass, and the closest town to each bearing within a 3–10 km radius of the case’s town was selected (or the location where the case animal was ill or died of anthrax, if different from the hometown). Once in the selected town center, each control animal was selected using the same two-stage sampling used for the village controls. The radius was extended to 20 km if no towns existed within 3–10 km and when there were no villages with the same species as the case animal. Inclusion criteria for “Geographic Area” controls were the same as the “Village” controls with the omission of the criterion “presence in the case’s village”.

#### Confidentiality and ethical considerations

Records associated with Case and Control participants were assigned a unique, anonymous Study ID; all records were maintained by the NFA in locked, secured locations. A verbal consent was obtained from all participants at the beginning of the survey. At the end of the investigation, all records were de-identified, and the linkages were destroyed. The investigation was reviewed and approved by the Chief Veterinary Officer (CVO) and Deputy Director of the Georgia NFA, Ministry of Agriculture and determined to be a part of routine investigation of anthrax case. Review by the NFA Department of Law for ethical and human subjects’ research consideration was not required. The investigation was additionally reviewed by the CDC National Center for Emerging and Zoonotic Infectious Diseases, Human Subjects Advisor and determined to be a non-research activity and therefore no additional approval was required.

#### Data collection

Field veterinarians were trained to administer the collaboratively developed survey tool in Georgian, Russian or Azerbaijani languages, which they pilot-tested to evaluate its reliability. Active enrollment of cases and controls started in October 2013. The first phase was a retrospective enrollment of those cases that occurred between 1 June 2013 and the study start date in October 2013. Once a case was identified, the owner and/or caretaker were invited to participate in the study. Individuals with a matched animal that met the control inclusion criteria were later identified and invited to participate. Upon receipt of informed consent, we administered the survey to the livestock owner or caretaker.

The survey contained sections regarding the clinical course and signs of disease relevant to the cases, and sections relevant to both groups. The survey collected information about the case and control animals’ exposure and risk factors within one-month prior to the disease onset of the case animal (or matched case for the controls). The survey questionnaire is available from the senior author upon request.

The residence location and phone number of the respondent were recorded on a removable cover sheet of the survey to follow up for clarification if needed and was destroyed after all data were gathered.

#### Measures

The data on the potential risk and exposure factors which may contribute to the occurrence of anthrax in livestock in Georgia, included: prior and frequency of anthrax vaccination as a protective factor; type of pasture used for grazing; pasture conditions; the use of supplemental food sources other than grazing pastureland; regions of the country and lands used; irrigation and livestock watering sources; type of regular veterinary monitoring of livestock health; and type of preventive anthrax measures; degree of awareness of disease and its ecology/epidemiology among livestock owners, livestock caretakers, and veterinarians; presence of anthrax cases on neighboring pastures or farms, or on farms or pastures in the area; animal demographics such as gender, age, nutritional status and condition prior to onset of illness in the individual susceptibility to anthrax; and season and seasonal migration of livestock within the sampled premises.

The “Village” control evaluates exposure and risk factors at the individual animal or owner level. Such factors may include: animal gender, age, nutritional status and conformation, vaccination history, and previous illness history.

The “Geographic Area” control evaluates the impact of exposure and risk factors that may be related to herd management practices, which influence livestock anthrax risk. This group was included because of its proximity to the “Case AMU” and thus should have a similar level of anthrax risk but was outside of the maximum distance of a vaccination intervention or surveillance zone surrounding the “Case Animal”.

#### Sample size and power estimates

The sample size for the study was anticipated to be 40 to 60 cases during the two years of study (20-30/year), and 80 to 120 controls for each of the two control groups, matched at a ratio of two controls per case. A matched 1:2 study design was capable of detecting a 20%-25% difference in level of exposure between cases and controls for n = 40 to 60 cases (odds ratios of 2.7 to 3.3), at the 0.2 level of exposure in controls, with a power of > = 80% (beta = 0.2) and 95% confidence (alpha = 0.05).

## Data management and statistical analyses

Data collection and secure data storage was performed by NFA in conjunction with the listed non-NFA authors. Trained personnel performed double data entry into Epi Info 7 (CDC, Atlanta, GA, USA) and exported into MS Excel for management and validation, and discrepancies in double entry were adjudicated. Analyses were conducted to estimate odds ratios and their 95% confidence Intervals for various exposures and risk factors in cases versus all controls [20, 21, 22]. A conditional logistic regression analysis for matched case-control design [23] was used to evaluate the odds of disease in the presence of the risk factor of interest. The analysis accounted for clustering if there are multiple cases and controls within the same AMU. In the univariate analysis, cases were separately compared to village controls, area controls, and to the combined control groups (all controls). Chi-square and Fisher’s exact tests (when appropriate) were used to identify associations between variables. The variables that met the criteria of p<0.25 in the univariate logistic models were entered into multivariable logistic models. In multivariable analysis, cases were compared to ‘all controls’ for analysis purposes. A step-wise selection of variables was performed to retain the variables that met the criteria of p<0.05 in the final model, to evaluate statistical significance. All statistical analyses were conducted using SAS v 9.4 (SAS Institute Inc., Cary, NC, USA).

## Results

During the study period, 36 anthrax cases met the case definition and were enrolled in the study; 67 matched village control animals and 71 matched area control animals were also recruited (Table 1). Cattle represented 83% of cases, followed by 14% sheep and 3% goats. Three cases had zero or one paired village control due to lack of matched species (sheep and goat) within the village. One sheep case had only one area control, as only one other sheep flock existed within the area control radius. Cases were not matched on sex or age; however, most of the case and control animals were female (86% and 95%, respectively).

**Table 1:** Characteristics of the enrolled case, village control, and area control animals.

Characteristic	Case	Case %	Village Control	Village Control %	Area Control	Area Control %
**TOTAL**	**36**		**67**		**71**	
Sex						
Female	31	86%	62	93%	69	97%
Male	5	14%	5	7%	2	3%
Species						
Cattle	30	83%	60	90%	60	85%
Sheep	5	14%	7	10%	9	13%
Goat	1	3%	0	0%	2	3%
Age (in years) (Mean, Range)						
Cattle	3.7 (1–9)		6.2 (1–16)		6 (1–16)	
Sheep	2.8 (2–4)		2.6 (0.67–5)		4.3 (1–11)	
Goat					2.8 (2–3.7)	

**Fig 1:**
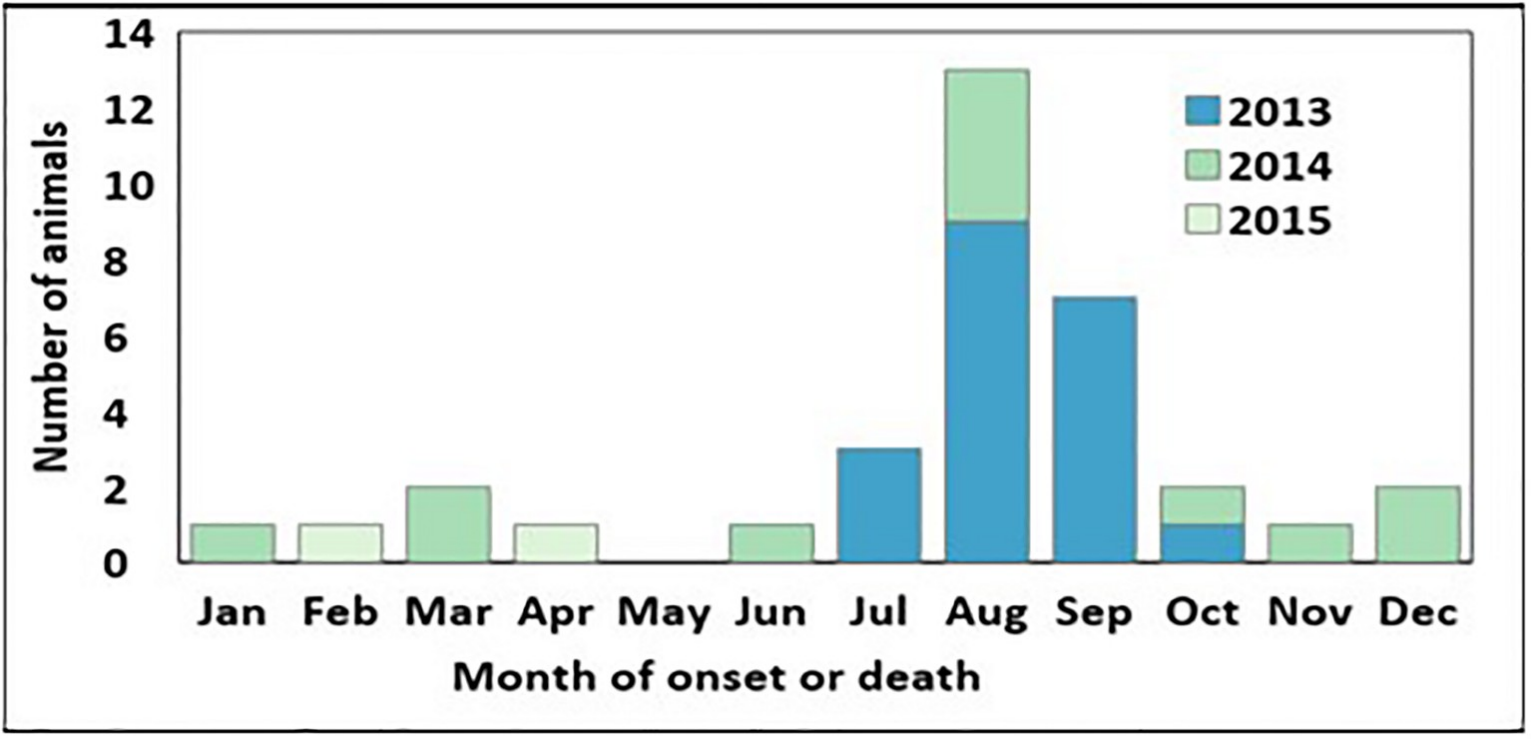
Case distribution during the two-year study period (1 June 2013–31 May 2015) by month and year of onset.

The majority of cases occurred in 2013 (n = 22, 61%); 12 cases (33%) were confirmed and enrolled in 2014, and 2 cases (6%) in 2015 (Fig 1). Cases occurred sporadically throughout the year, but they peaked from August to October.

Due to the small sample size of sheep and goats, only cattle were included in the statistical analyses. Thus, the findings from the analysis are applicable to cattle as the susceptible species.

Findings from the univariate analysis are in Table 2. The livestock that had an ear tag showed a significantly lower odds of anthrax (Odds Ratio [OR]: 0.21, 95% confidence interval [CI]: 0.077–0.56) than those with no individual ear tag, as did females (OR: 0.27, 95% CI: 0.08–0.96) when compared to males. Of female cattle, heifers had a significantly higher odds of anthrax (OR: 4.59, 95% CI: 1.04–20.29) when compared to pregnant females. The livestock vaccinated against anthrax at any point during their lifetime had a significantly lower odds of anthrax (OR: 0.05, 95%: 0.01–0.2) than those that were never vaccinated; the same held true for receiving any vaccine in the previous six months. However, recent treatment for parasites was not significant.

**Table 2:** Factors potentially associated with anthrax by univariate conditional logistic regression analysis: Cases vs all controls.

Factors	Case	All Controls	Odds Ratio (95% Confidence Limits)	P-value
**TOTAL**	**30**	**120**		
Ear Tag: Yes vs. No	10	83	0.21 (0.08–0.56)	**0.002**
Female vs. Male	25	114	0.27 (0.08–0.96)	**0.043**
Status of female (compared to pregnant)				
Pregnant	10	46	reference	
Milking	5	51	0.35 (0.08–1.45)	0.15
Dry	2	6	1.53 (0.25–9.27)	0.64
Heifer	8	9	4.59 (1.04–20.29)	**0.045**
Fed differently from herd	5	1	19.14 (2.23–164.04)	**0.007**
Housed differently from herd	3	3	3.81 (0.77–18.94)	0.10
Ever vaccinated against anthrax	2	67	0.05 (0.01–0.24)	**0.0002**
Vaccinated against any disease in last 6 months: Yes vs. No	15	100	0.10 (0.03–0.34)	**0.0002**
Treated for parasites	18	79	0.93 (0.31–2.73)	0.89
Another animal belonging to same owner died during Period 1*	4	4	4.70 (1.03–21.39)	**0.045**
Another animal in AMU but belonging to different owner died during Period 1	4	8	2.37 (0.48–11.79)	0.29
Anthrax burial site ≤1km from pasture	1	6	0.57 (0.06–5.04)	0.61
Water source: Permanent pond or lake	5	10	2.53 (0.69–9.24)	0.16

*Period 1: the 1 month before the date of onset or death of the case animal

There were significantly higher odds of anthrax during Period 1 compared to all controls (OR: 4.70, 95% CI: 1.03–21.39) (Table 2), when another livestock from the same owner experienced death in the past. However, cases did not have elevated odds of anthrax if another livestock in the same AMU but belonging to a different owner died during Period 1 (OR: 2.37, 95% CI: 0.48–11.79). Of the deaths that were reported in the AMU during Period 1, 4 of 6 were reported to be due to anthrax by case owners, and 6 of 11 by village controls. Area control respondents did not report any deaths due to anthrax during Period 1.

Most livestock (100% of cases and 97% of controls) in the study grazed on mixed grasses. Animals that were fed differently from other cattle belonging to the same owner had a significantly higher odds of anthrax (OR: 19.14, 95% CI: 2.23–164.03) than those fed the same. Case animals that shared a common feeding place with other animals at the village had a lower odds of anthrax infection compared to controls (village control OR: 0.24, 95% CI: 0.071–0.79, area control OR: 0.26, 95% CI: 0.09–0.79 and all controls OR: 0.24, 95% CI: 0.09–0.65) (Table 3). The location of the herd, including grazing on local land or pasture, in a covered fenced area or barn, or in an open fenced area, was not associated with anthrax outbreaks.

**Table 3:** Factors associated with anthrax in univariate conditional logistic regression analysis: Cases vs village, area and all controls.

Factors	Case	Area Controls (AC)	Village Controls (VC)	All Controls (All)	Odds Ratio (95% CI)	P-value
**TOTAL**	**30**	**60**	**60**	**120**		
Grazing on local pasture	15	38	38	76	VC: 0.14 (0.017–1.23)	0.076
					All: 0.32 (0.096–1.04)	0.059
Shared common feeding place	18	51	48	99	AC: 0.26 (0.088–0.79)	**0.017**
					VC: 0.24 (0.071–0.79)	**0.019**
					All: 0.24 (0.088–0.65)	**0.0052**
Migration route ≤1km from pasture	11	12	13	25	All: 2.76 (1.02–7.46)	**0.046**
Earthworks ≤1km from pasture	7	6	2	8	AC: 4.38 (0.82–23.37)	0.083
					VC: 9.52 (1.098–82.57)	**0.041**
					All: 5.52 (1.31–23.33)	**0.020**
Some or all animals migrated seasonally	4	8	7	15	VC: 7.13 (0.79–64.38)	0.081
					All: 3.20 (0.887–11.68)	0.078
Scavengers or predators present	14	37	31	68	AC: 0.37 (0.13–1.03)	0.057
					VC: 0.68 (0.20–2.35)	0.54
					All: 0.49 (0.19–1.28)	0.15
Presence of bloodsucking insects	13	23	25	48	AC: 1.18 (0.36–3.92)	0.78
					VC: 1.36 (0.36–5.17)	0.66
					All: 1.26 (0.40–4.01)	0.69
Herd received any veterinary care 6 months before Date 1*	15	46	40	86	AC: 0.20 (0.05–0.77)	**0.019**
					VC: 0.37 (0.11–1.25)	0.11
					All: 0.27 (0.087–0.81)	**0.02**
Veterinarians gave anthrax vaccine to any livestock in village in the 2 years before Date 1	9	29	29	58	AC: 0.23 (0.05–0.97)	**0.046**
					VC: 0.14 (0.024–0.81)	**0.028**
					All: 0.17 (0.039–0.78)	**0.023**

*Date 1: date of onset or death of case animal

AC = Area Controls; VC = Village Controls

The case’s herds that grazed on pasture within one kilometer of a migration route had a greater odds of anthrax (OR: 2.76, 95% CI: 1.02–7.46) compared to control’s herds. Insufficient respondents reported taking their livestock on seasonal migration to other pastures during Period 1, thus analysis could not be performed. However, using seasonal grazing land increased the chance of anthrax at a borderline significance (all controls OR: 3.20, 95% CI: 0.88–11.68 and village control OR: 7.12 (0.79–64.38).

Presence of scavenger or predator animals around the herd was not associated with anthrax when compared to village or area controls; however, the finding was at borderline significance (p = 0.057) compared to the latter group. Presence of bloodsucking insects was not associated with anthrax. Earthworks within one kilometer of the pasture significantly increased the odds of anthrax in livestock compared to village controls (OR: 9.52, 95% CI: 1.09–82.57) and all controls (OR: 5.52, 95% CI: 1.31–23.33), but at a borderline significance compared to the area control group (OR: 4.38, 95% CI: 0.82–23.37).

The odds of anthrax in herds receiving veterinary care for any reason six months before Date 1 was lower compared to all controls (OR: 0.27, 95% CI: 0.09–0.81) and area controls (OR: 0.20, 95% CI: 0.05–0.77). Also, the chance of anthrax was lower when the veterinarians vaccinated livestock in the village against anthrax in the two years before Date 1 compared to all controls (OR: 0.17, 95% CI: 0.04–0.78), area controls (OR: 0.23, 95% CI: 0.05–0.97) and village controls (OR: 0.14, 95% CI: 0.02–0.81). Females were significantly more likely to be vaccinated than males, and FMD vaccine was the only vaccine positively associated with receipt of the anthrax vaccine. Finally, a positive association was found between the responses for questions on ‘ever vaccinated against anthrax in lifetime’ and ‘did a veterinarian vaccinate your livestock against anthrax in the two years before Period 1’.

Twenty factors were qualified to be included into the multivariable logistic model using the criterion of p<0.25. Due to significant associations and correlations among some of the factors, three models were explored with some common factors among the models. The multivariable models compared cases to all controls. A total of five factors were included in the final three separate models (Table 4). The findings from model 3 demonstrate that vaccination within the last two years significantly reduced the odds of anthrax in cattle. The factor that was significantly protective against anthrax in all three final models was ‘female livestock being pregnant, or milking compared to heifer’. ‘Livestock being in covered fence area/barn’ was significantly protective in model 1. The only factor that significantly increased the odds of anthrax in models 1 and 3 was ‘livestock being fat compared to being normal’. The factor that showed a tendency towards significance (at p<0.1) was ‘source of water’. River as source of water was found to be protective in model 1 and pipe water was found to increase the odds of anthrax in cattle in model 3 at a borderline significance of p<0.1.

**Table 4:** Final multivariable conditional logistic regression models (Comparing cases to all controls).

Factors	Categories	Model 1		Model 2		Model 3	
		Odds Ratio (95% CI)	P-value	Odds Ratio (95% CI)	P-value	Odds Ratio (95% CI)	P-value
Did veterinarians vaccinate any livestock in your village against anthrax in the 2 years before DATE 1*?	Yes vs. No	0.053 (0.002, 1.35)	0.075	0.154 (0.01, 2.15)	0.16	0.014 (<0.001, 0.99)	**0.049**
	Don’t know vs. No	0.247 (0.009, 6.70)	0.41	0.351 (0.02, 6.30)	0.48	2.27 (0.14, 37.51)	0.57
**Where were all of your animals [same species as case] during PERIOD 1? No vs Yes**	Covered fenced area/barn	0.065 (0.005, 0.83)	**0.036**			0.32 (0.06, 1.74)	0.19
	Local grazing land / pasture			9.214 (0.58, 147.19)	0.12		
**What is the source of water on the pastureland that your herd had access to during PERIOD 1? No vs Yes**	River	0.094 (0.006, 1.44)	0.089	0.13 (0.01, 1.49)	0.10		
	Piped water					23.19 (0.59, 914.01)	0.09
What was the condition of the animal at DATE 1*?	Thin vs. normal	<0.001 (<0.001, >999.99)	0.99	<0.001 (<0.001, >999.99)	0.99	<0.001 (<0.001, >999.99)	0.99
	Fat vs. normal	38.48 (1.88, 788.84)	**0.018**	6.104 (0.74, 50.61)	0.09	32.43 (1.63, 646.69)	**0.023**
**Female Status**	Pregnant vs. Heifer	0.006 (<0.001, 0.73)	**0.037**	0.016 (<0.001, 0.62)	**0.027**	0.011 (<0.001, 0.95)	**0.047**
	Milking/Dairy vs. Heifer	0.003 (<0.001, 0.33)	**0.016**	0.01 (<0.001, 0.27)	**0.006**	0.004 (<0.001, 0.38)	**0.017**
	Dry vs. Heifer	0.59 (0.002, 153.41)	0.86	0.038 (<0.001, 8.40)	0.24	0.41 (0.007, 25.95)	0.68

*Date 1: date of onset or death of case animal

## Discussion

This matched case-control study in livestock was conducted from June 2013 to May 2015 in the country of Georgia as a follow-up to a human anthrax outbreak investigation in 2012 [6]. The study was strategically designed to identify those exposures and risk factors that contribute to the occurrence of livestock anthrax in the five most heavily affected regions in Georgia. The finding from the study could be used to optimize prevention and control strategies, including vaccination of livestock and educational campaigns of livestock owners. Our secondary goal was to identify gaps in the identification, notification, and reporting of livestock anthrax cases to strengthen the national anthrax surveillance system including case investigation.

The study design uniquely compared case animals to village controls as well as area controls to examine the risk factors at different proximal levels. The questionnaire was pilot-tested among owners with livestock that died of anthrax before the study period to reduce the potential bias of questions themselves. The pilot-testing helped to refine the questions and language used to reduce ambiguity. Some respondents were clearly influenced by local veterinarians, who assisted the study team in identifying the cases. In some circumstances, these veterinarians did want to interject and influence respondents during certain questions, particularly those about veterinary care received. To counteract this potential influence, the study team attended all the interviews as observers and thereby succeeded in dissuading the local veterinarians from interjecting with the help of staff of CDC and NFA.

“Vaccination” stood out to be a significant factor in reducing the risk of anthrax in livestock. Whilst this finding could be anticipated in developed countries, it was of great importance for the policy makers, veterinary service, and livestock owners in Georgia as the country develops a National Animal Health Program and has to justify national strategies to prevent and control specified priority diseases. Our study found various other factors to be significantly associated with anthrax through univariate and multivariable analysis. Anthrax can affect all mammals but is most commonly seen in grazing herbivore (livestock and/or wildlife) that presumably acquire infection by consuming *B*. *anthracis* spores along with contaminated vegetation or soil [7]. During 2007, there was a shift of responsibility for livestock vaccinations onto livestock owners, thus leading to a decrease in vaccinations administered and a resulting increase in livestock cases [6]. National programs have resulted in a global reduction of anthrax, although this is counteracted by the failure of more recent generations of veterinarians and farmers to recognize and report the disease, through lack of experience and the abandonment of vaccination in some places [1]. Control of anthrax begins with control of the disease in livestock, and vaccination of livestock has long been the hub of control programs [1, 24].

Timing of vaccination has shown significant association with the occurrence of subsequent deaths on case farms in the midst of an outbreak, the odds being significantly higher when vaccinated more than 1 week after the index case than on case farms where vaccination occurred within 1 week of the index case [7]. In the face of an outbreak, vaccination of affected herds was shown to reduce mortality rates beginning 8 days after vaccine administration [25]. Mongoh et al. (2008) [16] indicated that a second booster should be given during the outbreak period to protect livestock during the next outbreak, as a single dose of vaccination was not sufficient to elicit and maintain protective immunity. Our study did not evaluate those associations.

Veterinarians contribute a major role in building and implementing disease control programs in livestock. Veterinary care in the previous six months as well as veterinarians’ involvement in vaccinating livestock against anthrax in the previous two years were found to be significant in lowering the risk of anthrax in our study. The livestock vaccinated against anthrax at any point during their lifetime had significantly lower odds of anthrax than those that were never vaccinated. The same held true for receiving any vaccine in the previous six months. In a study in various livestock species in North Dakota, Mongoh et al. (2008) [16] indicated that agricultural regions with inadequate veterinary public health services have the highest reported occurrence of anthrax.

Other vaccinations may also influence the administration of anthrax vaccination as the livestock owners may find it feasible to administer multiple vaccinations at the same time. In our study, administration of FMD vaccination was highly associated with the administration of anthrax vaccination, probably because of governmental supply of FMD vaccination. Hence, owners chose to vaccinate for anthrax during the FMD vaccination. This also reflects on responsible ownership. Another indicator of responsible ownership was ear tagging of livestock, which again reduced the odds of anthrax; females were more likely to be ear tagged and hence were more likely to be vaccinated and protected against anthrax. Female animals being the productive unit would be invested with more preventive care due to their long-term benefits. The majority of the livestock owners keep female animals, and there are few beef farmers in the country. Furthermore, female pregnant livestock were protected from anthrax when compared to heifers. Younger females were more likely to have anthrax because they missed the vaccine cycle due to young age during previous vaccination campaigns. WHO (2008) [1] stated that sex is one of factors that may influence the incidence of the disease at any one site. However, in a study of Canadian wood bison, there were higher numbers of mature bulls affected consistently, and it was suggested that behavioral stress factors associated with the late summer rut may have predisposed the bulls to infection [26]. Higher attack rates in males have also been reported previously in cattle anthrax outbreaks [16, 25].

The odds of anthrax were found to be higher in this investigation when another animal belonging to the same owner died during Period 1; however, the disease was not likely if the other dead livestock belonged to a different owner in the same AMU. This finding may reflect upon individual owner practices and preventive care. Other studies have also found significant associations of the disease with presence of other deaths in nearby premises [16]. Similarly, presence and type of predators on the pasture were reported in that study to be associated with the disease [16]. In our study, we only saw a trend towards significance of disease with presence of scavenger or predator animals around the herd. In our study, the presence of bloodsucking insects did not seem to impact the disease presence. Previous studies have suggested that biting flies may act as mechanical vectors [11], whereas necrophilic flies or blowflies may contaminate vegetation after feeding on contaminated carcasses and thereby may contribute to the disease transmission and amplification of outbreaks [10, 16, 27].

Feeding patterns and sharing pasture among grazing livestock play a role in transmission of diseases such as anthrax. It is a long-held belief that livestock generally acquire anthrax by ingestion of spores while grazing or browsing [1]. Anthrax was noted in herds that grazed on pasture within 1 kilometer of a migration route when compared to herds that grazed farther from migration routes, and also when ground disturbance due to earthworks happened within 1 kilometer of the pasture. Using a seasonal grazing land tended to increase the odds of anthrax. The density of livestock on pasture has been previously reported to influence the incidence of disease, and seasonal pastures may have higher concentrations of livestock relative to other areas [7]. Moreover, seasonal pastures are used by and concentrate herds from many different areas, with different herd management practices and different regional risk of anthrax to the herd. There may be a history of disease in livestock brought to seasonal pasture and dying from anthrax either en route, along a migration route, or after arrival at the seasonal pasture; this potentiality was anecdotally shared by several veterinarians who assisted the study investigators. The association of anthrax with the livestock being fat as well as use of piped water could be related to a finding specific to the cases in this investigation, but no biologically plausible explanations for these findings are apparent at the time of this study. The disease in our study however, was not affected by the location of the herd, including grazing on local land or pasture, or in an open fenced area, although multivariable analysis revealed that a covered fenced area or barn was significantly protective. On the contrary, sharing a common feeding place with other livestock in the village was found to be protective of the disease in univariate analysis. This may be reflective of the livestock in the village AMU being subject to some shared, protective management practices on the part of the herders caring for the livestock as a group. However, this may require further investigation to examine the counterintuitive finding.

As with many studies, our study had some limitations. These include the retrospective aspect of data collection, small sample size, concurrent prophylactic vaccination campaigns, dependency on reported cases instead of recruitment through direct measure of the disease, potential recall bias (particularly in controls due to no specific event to remember); and the presence of local veterinarians during the surveys, which might have influenced participant responses.

## Conclusions

The control strategies that were recommended for anthrax included a combination of vaccination, quarantine, and proper carcass handling and disposal. Overall, the information obtained from this study has involved and been presented to decision makers, used to build technical capacity of regional and national veterinary staff, and fostered a One Health approach to the control of zoonotic diseases like anthrax, which will optimize prevention and control strategies. For example, a multi-agency anthrax One Health team was established to investigate cases and co-develop educational materials for farmers.

The investigation process involved a series of trainings and workshops for participants and stakeholders to promote an understanding of epidemiological investigations and the economics of disease control with anthrax as a model. Georgia now has embedded the knowledge and specific evidence that vaccination is a highly protective measure to prevent anthrax deaths among livestock. Hence, primary emphasis for disease prevention will be given to vaccination, with a specific mark/tag for vaccination being desirable. Alternatively, a formal vaccination record given to the owner, or livestock registration is recommended. Education of livestock keepers in Georgia on the importance of vaccination is an overriding priority. Vaccination teams can play an increased role with more attention paid to delivery of standard memorable messages at the time of vaccination and to disseminating public announcements. It is overwhelmingly the case that vaccination of livestock against anthrax is protective and is an effective risk mitigation for anthrax in Georgia.

## Supporting information

S1 Questionnaire (DOCX)

S2 Questionnaire (DOCX)

S1 Data (XLSX)
