# Supplementary material for: Risk factors associated with the occurrence of anthrax outbreaks in livestock in the country of Georgia: A case-control investigation 2013-2015
Source: PLoS One. 2019 May 2;14(5):e0215228. doi: 10.1371/journal.pone.0215228 (PMC6497231; doi:10.1371/journal.pone.0215228)
Supplement: S1 Questionnaire — (DOCX) [file pone.0215228.s001.docx]

**Animal Anthrax Investigation Questionnaire**

___ **Case** ___ **Village Control** ___ **Area Control**

**Name of Interviewer:** ___________________________________________

**Date of Interview (dd/mm/yy):** _______________________

**Time Interview Began:** _____________________

**INTRODUCTION**

Good Morning/Afternoon,

I am …… [name, surname] …….. and …… [name, surname] …… are my colleagues and we are the representatives of the National Food Agency of the Ministry of Agriculture of Georgia.

I would like to tell you that in Georgia within the “National Animal Health Program” the Animal Anthrax investigation is being carried out by NFA. The objective of the investigation is to improve and develop Anthrax prevention and control in Georgia.

CONTROL: We would like to ask you several questions about your [same species as case:______________]. Did you own a [same species as case] during PERIOD 1?

[***investigator writes the species of the case animal, all control animals are the same species as case animal***]

[***investigator explains the PERIOD 1 as the 30 days before DATE 1***]

[***If respondent did not have the same species of animal during PERIOD 1***]:

Thank you for your time. Because we must ask questions about [same species as case], we do not need to take up any more of your time. Have a nice day.

CASE: The questions concern the [*species of case*] that had anthrax on ………….. [date] and other domestic animals as well.

Our interview will continue for about 45 minutes. The interview is confidential; all the data collected will be used for this investigation only. During the interview if you decide to no longer participate in the investigation, we will stop the interview. Please, take into account that your active and sincere participation will contribute much to the effective prevention and improved control of anthrax in Georgia.

If you don’t mind we can start the interview.

Do you agree to participate?

Respondent 1 Respondent 2

## ___ Yes ___ No ___ Yes ___ No

**Respondents**

1. What is your name? [Respondent 1]:________________________________

___ Owner ___ Shepherd/Animal Handler

- 1. Phone number: ________________________________

1. What is your name? [Respondent 2]:______________________________

___ Owner ___ Owner’s family ___ Shepherd/Animal Handler

- 1. Phone number: ________________________________

**Part 1. ANIMAL OWNER / HANDLER**

**Respondent 1**

1. During PERIOD 1, which is the 30 days before [say the DATE 1 date], who cared for your [same species as case animal]?

___ Just me ___ Myself and my family member(s)

___ Only my family member(s) ___ Myself, my family members, and the shepherd

___ Myself and the shepherd ___ Only the shepherd

*[If answered ‘Just Me” go to Question 4]*

- 1. Since others also cared for the animal, are they available right now to participate in this interview? It would be very helpful to hear from them as well.

___ Yes

___ No

___ Don’t know

Since they are not available, we would like to collect their name, role and phone number so we can schedule an interview with them.

- 1. What is their name?

___________________________________

- 1. What is their role?

____________________________________

- 1. What is their phone number?

___________________________________

1. Generally, how much time did you care for the animal in PERIOD 1?

___ All the time ___ Morning and evening

___ During the day ___ Once every 2 days

___ Once per week ___ Once per month

___ No time during Period 1

- 1. How many years experience do you have caring for livestock animals? ______

1. How old are you (in years)? ______
2. gender?

___ Male ___ Female

**Respondent 2**

1. Generally, how much time did you care for the animal in PERIOD 1?

___ All the time ___ Morning and evening

___ During the day ___ Once every 2 days

___ Once per week ___ Once per month

___ No time during period

- 1. How many years experience do you have caring for livestock animals?? ______

1. How old are you (in years)? ______
2. gender?

___ Male ___ Female

**Part 2. CONDITION & HISTORY**

CASE: I am going to ask you some questions about the animal that had anthrax.

CONTROL: What was the last [*Case species animal*] that you worked with? Did you have or work with this animal during the 30 days before date 1? Now, I am going to ask you some questions about this animal.

1. Did the animal have an ear tag?

| Respondent 1 | Respondent 2 |
| --- | --- |
| ___ Yes  ___ No  ___ Don’t Know | ___ Yes  ___ No  ___ Don’t Know |

*[Go to Question 10.1]*

*[Go to Question 10.2]*

- 1. Do you rememeber what the number was?

| Respondent 1 | Respondent 2 |
| --- | --- |
| ___ Yes  ___ No | ___ Yes  ___ No |

*[Go to Question 10.2]*

*[Go to Question 10.3]*

- 1. What ws the number?

| Respondent 1 | Respondent 2 |
| --- | --- |
| _______________________________ | _________________________________ |

10..3 What was its name and color?

| Respondent 1 | Respondent 2 |
| --- | --- |
| _______________________________ | _________________________________ |

1. Where did you get the animal?

| Respondent 1 | Respondent 2 |
| --- | --- |
| ___ Born within herd  ___ Bought from within municipality  ___ Bought from outside municipality  ___ Bought from outside Georgia  ___ Other, specify________________ | ___ Born within herd  ___ Bought from within municipality  ___ Bought from outside municipality  ___ Bought from outside Georgia  ___ Other, specify________________ |

1. What was its sex:

| Respondent 1 | Respondent 2 |
| --- | --- |
| ___ Female  ___ Male | ___ Female  ___ Male |

*[Go to Question 12.1]*

*[Go to Question 12.2]*

- 1. *[If female]* On DATE 1, was the animal:

| Respondent 1 | Respondent 2 |
| --- | --- |
| ___ Pregnant  ___ Milking/Dairy  ___ Dry  ___ Heifer | ___ Pregnant  ___ Milking/Dairy  ___ Dry  ___ Heifer |

- 1. *[If male]* On DATE 1, was the animal:

| Respondent 1 | Respondent 2 |
| --- | --- |
| ___ Not castrated  ___ Castrated | ___ Not castrated  ___ Castrated |

1. What was estimated age at DATE 1?

| Respondent 1 | Respondent 2 |
| --- | --- |
| _____ *□* years  _____ *□* months  *□* Don’t know | _____ *□* years  _____ *□* months  *□* Don’t know |

1. What was the condition of the animal at DATE 1?

| Respondent 1 | Respondent 2 |
| --- | --- |
| ___ Thin  ___ Normal  ___ Fat  ___ Don’t Know | ___ Thin  ___ Normal  ___ Fat  ___ Don’t Know |

1. How long had you taken care of it at DATE 1?

| Respondent 1 | Respondent 2 |
| --- | --- |
| _____ ___ years ___ months  ___ Don’t know | _____ ___ years ___ months  ___ Don’t know |

1. Were there any changes to the animal in PERIOD 1?

| Respondent 1 | Respondent 2 |
| --- | --- |
| ___ Yes  ___ No  ___ Don’t Know | ___ Yes  ___ No  ___ Don’t Know |

*[If No or Don’t Know, go to Question 17]*

- 1. If yes, please select the changes

| Respondent 1 | Respondent 2 |
| --- | --- |
| ___ Reduced milk yield  ___ Abortion  ___ Off feed for more than 12 hours  ___ Fever (_____°C)  ___ Limping  ___ Difficulty breathing  ___ Other, specify__________________ | ___ Reduced milk yield  ___ Abortion  ___ Off feed for more than 12 hours  ___ Fever (_____°C)  ___ Limping  ___ Difficulty breathing  ___ Other, specify_______________________ |

1. Was there any difference in feeding the animal compared to the rest of all of your *[same species as case]* during PERIOD 1?

| Respondent 1 | Respondent 2 |
| --- | --- |
| ___ Yes  ___ No  ___ Don’t Know  ___ No other animals to compare to | ___ Yes  ___ No  ___ Don’t Know  ___ No other animals to compare to |

*[If No or Don’t Know, go to Question 18]*

*[Go to Question 19]*

- - 1. If yes, describe

| Respondent 1 | Respondent 2 |
| --- | --- |
| ___________________________________ | ___________________________________ |

1. Was the animal housed differently compared to the rest of all of your *[same species as case]* during PERIOD 1?

| Respondent 1 | Respondent 2 |
| --- | --- |
| ___ Yes | ___ Yes |
| ___ No | ___ No |
| ___ Don’t Know | ___ Don’t Know |

*[If No or Don’t Know, go to Question 19]*

- - 1. If yes, describe:

| Respondent 1 | Respondent 2 |
| --- | --- |
| ___________________________________ | ___________________________________ |

1. Was the animal ever vaccinated against anthrax during it’s lifetime?

| Respondent 1 | Respondent 2 |
| --- | --- |
| ___ Yes | ___ Yes |
| ___ No | ___ No |
| ___ Don’t Know | ___ Don’t Know |

*[If No or Don’t Know, go to Question 20]*

- 1. If yes, how many times was it vaccinated against anthrax?

| Respondent 1 | Respondent 2 |
| --- | --- |
| ___________________________________ | ___________________________________ |

- 1. When was the last time it was vaccinated against anthrax before Date 1?

| Respondent 1 | Respondent 2 |
| --- | --- |
| ___ Less than 1 month before date 1___ 1-6 months before date 1  ___ 6-12 months before date 1  ___ More than 12 months before date 1 | ___ Less than 1 month before date 1  ___ 1-6 months before date 1  ___ 6-12 months before date 1  ___ More than 12 months before date 1 |

1. Was the animal vaccinated against any disease other than anthrax in the six months before Date 1?

| Respondent 1 | Respondent 2 |
| --- | --- |
| ___ Yes | ___ Yes |
| ___ No | ___ No |
| ___ Don’t Know/Don’t remember | ___ Don’t Know/ Don’t remember |

*[Go to Question 21].*

- 1. If yes, list the diseases and the date vaccine was last given for each:

| Respondent 1 | | Respondent 2 | |
| --- | --- | --- | --- |
| Diseases | Date last given (mm/yy) | Diseases | Date last given (mm/yy) |
| ___ FMD |  | ___ FMD |  |
| ___ Pasteurellosis |  | ___ Pasteurellosis |  |
| ___ Brucellosis |  | ___ Brucellosis |  |
| ___ Bradzot (Clostridium) |  | ___ Bradzot (Clostridium) |  |
| ___ Other  ________________ |  | ___ Other  ______________ |  |

1. .2 t age unit] Was the animal treated for ecto- or endo-parasites inthe six months before Date 1?

| Respondent 1 | Respondent 2 |
| --- | --- |
| ___ Yes | ___ Yes |
| ___ No | ___ No |
| ___ Don’t Know/Don’t remember | ___ Don’t Know/ Don’t remember |

*[Go to Question 22].*

21.1. If yes, what was the date of the treatment during that time

| Respondent 1 | Respondent 2 |
| --- | --- |
| Date last given (mm/yy) | Date last given (mm/yy) |
|  |  |

**Part 3. CLINICAL INFORMATION [Only ask for case animals]**

1. What was the outcome of the anthrax? [ask each outcome listed below]

| Respondent 1 | Respondent 2 |  |
| --- | --- | --- |
| ___ Sick  ___ Slaughtered after being sick  ___ Slaughtered without symptom of sickness  (confirmed after slaughtered)  ___ Butchered after dead  ___ Recovered  ___ Dead  ___ Sold, final outcome unknown | ___ Sick  ___ Slaughtered after being sick  ___ Slaughtered without symptom of sickness  (confirmed after slaughtered)  ___ Butchered after dead  ___ Recovered  ___ Dead  ___ Sold, final outcome unknown | |

- 1. What was the duration of illness from onset of illness until outcome?

| Respondent 1 | Respondent 2 |
| --- | --- |
| ___ Found dead  ___ Less than half a day  ___ Between half a day and one day  ___ Greater than 24 hours | ___ Found dead  ___ Less than half a day  ___ Between half a day and one day  ___ Greater than 24 hours |

- 1. If more than one day, give the number of days

| Respondent 1 | Respondent 2 |
| --- | --- |
| ________ | __________ |

1. I am going to ask you about a number of signs of sickness you might have observed in your animal, either when alive or dead. After I read each observation, please say “yes”, “no’, or “don’t know”.

*[For questions specifically about an animal that has died, if the animal is not dead check “Not Applicable”]*

| **Clinical signs** | Respondent 1 | Respondent 2 |
| --- | --- | --- |
| Fever | ___ Yes ___ No ___ Don’t Know | ___ Yes ___ No ___ Don’t Know |
| Off feed | ___ Yes ___ No ___ Don’t Know | ___ Yes ___ No ___ Don’t Know |
| Diarrhea | ___ Yes ___ No ___ Don’t Know | ___ Yes ___ No ___ Don’t Know |
| Convulsion | ___ Yes ___ No ___ Don’t Know | ___ Yes ___ No ___ Don’t Know |
| Muscle tremors | ___ Yes ___ No ___ Don’t Know | ___ Yes ___ No ___ Don’t Know |
| Difficulty breathing | ___ Yes ___ No ___ Don’t Know | ___ Yes ___ No ___ Don’t Know |
| Fallen down suddenly | ___ Yes ___ No ___ Don’t Know | ___ Yes ___ No ___ Don’t Know |
| Swelling  *[If yes, select swollen body part(s)]* | ___ Yes ___ No ___ Don’t Know | ___ Yes ___ No ___ Don’t Know |
|  | ___ Tongue ___ Throat ___ Sternum  ___ Flanks ___ Perineum  ___ Other:____________________ | ___ Tongue ___ Throat ___ Sternum  ___ Flanks ___ Perineum  ___ Other:___________________ |
| Drop in milk production | ___ Yes ___ No ___ Don’t Know  ___ Not applicable | ___ Yes ___ No ___ Don’t Know  ___ Not applicable |
| Discolored milk (Color____________) | ___ Yes ___ No ___ Don’t Know  ___ Not applicable | ___ Yes ___ No ___ Don’t Know  ___ Not applicable |
| Sudden death (within 8 hours of onset of clinical symptoms) | ___ Yes ___ No ___ Don’t Know | ___ Yes ___ No ___ Don’t Know |
| Found dead | ___ Yes ___ No ___ Don’t Know | ___ Yes ___ No ___ Don’t Know |
| Rapid bloating of the dead animal | ___ Yes ___ No ___ Don’t Know  ___ Not applicable | ___ Yes ___ No ___ Don’t Know  ___ Not applicable |
| Lack of stiffness of the body after death | ___ Yes ___ No ___ Don’t Know  ___ Not applicable | ___ Yes ___ No ___ Don’t Know  ___ Not applicable |
| Blood from the carcass did not clot | ___ Yes ___ No ___ Don’t Know  ___ Not applicable | ___ Yes ___ No ___ Don’t Know  ___ Not applicable |
| Dark/Tarry colored blood observed | ___ Yes ___ No ___ Don’t Know  ___ Not applicable | ___ Yes ___ No ___ Don’t Know  ___ Not applicable |
| Bloody discharge from carcass | ___ Yes ___ No ___ Don’t Know  ___ Not applicable | ___ Yes ___ No ___ Don’t Know  ___ Not applicable |
| Swollen spleen of the dead animal | ___ Yes ___ No ___ Don’t Know  ___ Not applicable | ___ Yes ___ No ___ Don’t Know  ___ Not applicable |
| Other: Specify_________________  _____________________________ | ___ Yes ___ No ___ Don’t Know | ___ Yes ___ No ___ Don’t Know |

**Part 4. HERD DEMOGRAPHY** [ask for both animals and controls]

I am going to ask you several questions about the group of animals that the specific animal we are talking about spends time with during PERIOD 1. This includes animals that were pastured, fed, or travelled together, and can include animals that belong to several different people. I will call this group a HERD. Do you understand?

1. How many total animals, by species, were there in the herd during PERIOD 1? How many of those animals became sick and recovered, are still sick, and how many animals died? Please indicate for each species.

|  | Respondent 1 | | | | | Respondent 2 | | | | |
| --- | --- | --- | --- | --- | --- | --- | --- | --- | --- | --- |
| Species | Total | Normal | Sick and recovered | Still sick | Dead | Total | Normal | Sick and recovered | Still sick | Dead |
| Cattle |  |  |  |  |  |  |  |  |  |  |
| Goats |  |  |  |  |  |  |  |  |  |  |
| Sheep |  |  |  |  |  |  |  |  |  |  |
| Horses |  |  |  |  |  |  |  |  |  |  |
| Pigs |  |  |  |  |  |  |  |  |  |  |
| Other: specify ____________________ |  |  |  |  |  |  |  |  |  |  |

- 1. How many other owners owned those animals?

| Respondent 1 | Respondent 2 |
| --- | --- |
| ________________ | _________________ |

1. Where were all of your animals [same species as case] during PERIOD 1(check all that apply)?

| Respondent 1 | Respondent 2 |
| --- | --- |
| □ Covered fenced area/barn  □ Local grazing land / pasture  □ Fenced area  □ Seasonal grazing land  □ Other (Specify ________________) | □ Covered fenced area/barn  □ Local grazing land / pasture  □ Fenced area  □ Seasonal grazing land  □ Other (Specify ________________) |

- 1. If all of your animals [same species as case] were kept in more than 1 location during PERIOD 1, how many times during a week were they moved between locations?

| Respondent 1 | Respondent 2 |
| --- | --- |
| ___ Every day  ___ Most days  ___ Some days  ___ 1 day  ___ Don’t know  ___ Did not move | ___ Every dayDaily  ___ Most days  ___ Some days  ___ 1 day  ___ Don’t know  ___ Did not move |

**Go to 26**

- 1. How were they moved between locations?

| Respondent 1 | Respondent 2 |
| --- | --- |
| ___ Walked  ___ Transported by vehicle | ___ Walked  ___ Transported by vehicle |

- 1. Was the specific animal we are talking about moved between locations differently than the rest of your [same species as case] during PERIOD 1?

| Respondent 1 | Respondent 2 |
| --- | --- |
| ___ Yes | ___ Yes |
| ___ No | ___ No |
| ___ Don’t Know | ___ Don’t Know |

1. Did all of your animals that are the *[same species as case]* share a common feeding place with other animals at the village?

| Respondent 1 | Respondent 2 |
| --- | --- |
| ___ Yes | ___ Yes |
| ___ No | ___ No  *[Go to Question 27]* |
| ___ Don’t Know/Don’t remember | ___ Don’t Know/ Don’t remember |
| ___ Not applicable [Don’t move animals] | ___ Not applicable [Don’t move animals] |

- 1. If yes, where:

| Respondent 1 | Respondent 2 |
| --- | --- |
| ___ Common pastureland  ___ Common feeding trough  ___ Other _________________ | ___ Common pastureland  ___ Common feeding trough  ___ Other _________________ |

**Part 5. SEASONAL MIGRATION**

1. Does a migration route pass within 1 kilometer of the pasture where the herd grazed during PERIOD 1?

| Respondent 1 | Respondent 2 |
| --- | --- |
| ___ Yes  ___ No  ___ Don’t Know/Don’t remember | ___ Yes  ___ No  ___ Don’t Know/ Don’t remember |

1. Did you move all of your animals [*same species as case]* to or from seasonal grazing land (the summer pastures or winter pastures) away from home during PERIOD 1?

| Respondent 1 | Respondent 2 |
| --- | --- |
| ___ Yes, all animals  ___ Yes, but not all animals  ___ No  ___ Don’t Know | ___ Yes, all animals  ___ Yes, but not all animals  ___ No  ___ Don’t Know |

*[Go to question 28.1]*

*[Go to question 29]*

- 1. How were they moved?

| Respondent 1 | Respondent 2 |
| --- | --- |
| ___ Walked  ___ Transported by vehicle | ___ Walked  ___ Transported by vehicle |

- 1. Was the specific animal we are talking about moved to or from seasonal grazing land differently than the rest of your [same species]?

| Respondent 1 | Respondent 2 |
| --- | --- |
| ___ Yes | ___ Yes |
| ___ No | ___ No |
| ___ Don’t Know | ___ Don’t Know |

1. Do you ever use seasonal grazing lands?

| Respondent 1 | Respondent 2 |  |
| --- | --- | --- |
| ___ Yes  ___ No  ___ Don’t Know/Don’t remember | ___ Yes  *[If No or Don’t Know, go to Question 35]*  ___ No  ___ Don’t Know/ Don’t remember | |

1. Where is your seasonal grazing land located?

Region_______________________________

Municipality___________________________

Location ______________________________

Name of grazing place _____________________________

1. During which month do you normally leave home with all of your animals to go to the grazing land?

| Respondent 1 | Respondent 2 |
| --- | --- |
| Month: _________________  ___ Varies each year  ___ Don’t Know/Don’t remember | Month: _________________  ___ Varies each year  ___ Don’t Know/Don’t remember |

1. The last time you went to this seasonal grazing land, how long did it take to get there with all the [*same species as case*] animals (in days)?

| Respondent 1 | Respondent 2 |
| --- | --- |
| _________________  ___ Don’t Know/Don’t remember | _________________  ___ Don’t Know/Don’t remember |

1. The last time you went to this seasonal grazing land, how long did all your [*same species as case*] animals stay there (in days)?

| Respondent 1 | Respondent 2 |
| --- | --- |
| _________________  ___ Don’t Know/Don’t remember | _________________  ___ Don’t Know/Don’t remember |

1. The last time you went, how many, if any of the [*same species as case*] animals died when traveling to and from the seasonal grazing land?

| Respondent 1 | Respondent 2 |
| --- | --- |
| _________________  ___ Don’t Know/Don’t remember | _________________  ___ Don’t Know/Don’t remember |

**Part 6. HERD ANIMALS**

In the next few questions, I will ask you only about the animals that you owned during [PERIOD 1].

1. [CASE]:So, did any of your animals other than the animal that died of anthrax die suddenly during PERIOD 1?

[CONTROL]*:* So, did any of your animals die suddenly during PERIOD 1?

| Respondent 1 | Respondent 2 |
| --- | --- |
| ___ Yes | ___ Yes |
| ___ No | ___ No |
| ___ Don’t Know/Don’t remember | ___ Don’t Know/ Don’t remember |

*[If No or Don’t Know,*

*go to question 36]*

- 1. Was it determined that the animal died of anthrax?

| Respondent 1 | Respondent 2 |
| --- | --- |
| ___ Yes  ___ No  ___ Don’t Know/Don’t remember | ___ Yes  ___ No  ___ Don’t Know/ Don’t remember |

- 1. Who was involved in determining that the animal died of anthrax [check all that apply]?

| Respondent 1 | Respondent 2 |
| --- | --- |
| ___ Owner  ___ Caretaker of the animal  ___ Neighbor  ___ Local medical doctor  ___ Private veterinarian  ___ Laboratory  ___ Governmentveterinarian  ___ Other (specify) __________________ | ___ Owner  ___ Caretaker of the animal  ___ Neighbor  ___ Local medical doctor  ___ Private veterinarian  ___ Laboratory  ___ Government veterinarian  ___ Other (specify) _________________ |

- 1. What did you do [check all that apply]?

| Respondent 1 | Respondent 2 |
| --- | --- |
| ___ Separated and moved to a different location  ___ Gave all animals antibiotics  ___ Vaccinated animals against anthrax  ___ Disinfected holding area  ___ Nothing | ___ Separated and moved to a different location  ___ Gave all animals antibiotics  ___ Vaccinated animals against anthrax  ___ Disinfected holding area  ___ Nothing |

- 1. Did you observe the following in the animals that died?

After I read each observation, please say “yes”, “no’, or “don’t know”.

| Observations | Respondent 1 | Respondent 2 |
| --- | --- | --- |
| Fever | ___ Yes ___ No ___ Don’t Know | ___ Yes ___ No ___ Don’t Know |
| Off feed | ___ Yes ___ No ___ Don’t Know | ___ Yes ___ No ___ Don’t Know |
| Diarrhea | ___ Yes ___ No ___ Don’t Know | ___ Yes ___ No ___ Don’t Know |
| Convulsion | ___ Yes ___ No ___ Don’t Know | ___ Yes ___ No ___ Don’t Know |
| Muscle tremors | ___ Yes ___ No ___ Don’t Know | ___ Yes ___ No ___ Don’t Know |
| Difficulty breathing | ___ Yes ___ No ___ Don’t Know | ___ Yes ___ No ___ Don’t Know |
| Fallen down suddenly | ___ Yes ___ No ___ Don’t Know | ___ Yes ___ No ___ Don’t Know |
| Swelling  *[If yes, select swollen body part(s)]* | ___ Yes ___ No ___ Don’t Know | ___ Yes ___ No ___ Don’t Know |
|  | ___ Tongue ___ Throat ___ Sternum  ___ Flanks ___ Perineum  ___ Other:____________________ | ___ Tongue ___ Throat ___ Sternum  ___ Flanks ___ Perineum  ___ Other:___________________ |
| Drop in milk production | ___ Yes ___ No ___ Don’t Know | ___ Yes ___ No ___ Don’t Know |
| Discolored milk (Color____________) | ___ Yes ___ No ___ Don’t Know  ___ Not applicable | ___ Yes ___ No ___ Don’t Know  ___ Not applicable |
| Sudden death (within 8 hours of onset of clinical symptoms) | ___ Yes ___ No ___ Don’t Know | ___ Yes ___ No ___ Don’t Know |
| Found dead | ___ Yes ___ No ___ Don’t Know | ___ Yes ___ No ___ Don’t Know |
| Rapid bloating of the dead animal | ___ Yes ___ No ___ Don’t Know  ___ Not applicable | ___ Yes ___ No ___ Don’t Know  ___ Not applicable |
| Lack of stiffness of the body after death | ___ Yes ___ No ___ Don’t Know  ___ Not applicable | ___ Yes ___ No ___ Don’t Know  ___ Not applicable |
| Blood did not clot | ___ Yes ___ No ___ Don’t Know  ___ Not applicable | ___ Yes ___ No ___ Don’t Know  ___ Not applicable |
| Dark/Tarry colored blood observed | ___ Yes ___ No ___ Don’t Know  ___ Not applicable | ___ Yes ___ No ___ Don’t Know  ___ Not applicable |
| Bloody discharge from carcass | ___ Yes ___ No ___ Don’t Know  ___ Not applicable | ___ Yes ___ No ___ Don’t Know  ___ Not applicable |
| Swollen spleen of the dead animal | ___ Yes ___ No ___ Don’t Know  ___ Not applicable | ___ Yes ___ No ___ Don’t Know  ___ Not applicable |
| Other: Specify__________________  ______________________________ | ___ Yes ___ No ___ Don’t Know | ___ Yes ___ No ___ Don’t Know |

**Now I will ask you only about the animals that were pastured with your own animals, but were owned by others, during PERIOD 1.**

1. Did any of these animals die suddenly in PERIOD 1?

| Respondent 1 | Respondent 2 |
| --- | --- |
| ___ Yes  ___ No  ___ Don’t Know | ___ Yes  ___ No  ___ Don’t Know |

*[If No or Don’t Know, go to question 37]*

- 1. Was it determined the animal died of anthrax?

| Respondent 1 | Respondent 2 |
| --- | --- |
| ___ Yes  ___ No  ___ Don’t Know | ___ Yes  ___ No  ___ Don’t Know |

- 1. What did you do[check all that apply]?

| Respondent 1 | Respondent 2 |
| --- | --- |
| ___ Separated and moved to a different location  ___ Gave all animals antibiotics  ___ Vaccinated animals against anthrax  ___ Disinfected holding area  ___ Nothing | ___ Separated and moved to a different location  ___ Gave all animals antibiotics  ___ Vaccinated animals against anthrax  ___ Disinfected holding area  ___ Nothing |

- 1. Did you observe the following in the animals that died?

After I read each observation, please say “yes”, “no’, or “don’t know”.

| Observations | Respondent 1 | Respondent 2 |
| --- | --- | --- |
| Fever | ___ Yes ___ No ___ Don’t Know | ___ Yes ___ No ___ Don’t Know |
| Off feed | ___ Yes ___ No ___ Don’t Know | ___ Yes ___ No ___ Don’t Know |
| Diarrhea | ___ Yes ___ No ___ Don’t Know | ___ Yes ___ No ___ Don’t Know |
| Convulsion | ___ Yes ___ No ___ Don’t Know | ___ Yes ___ No ___ Don’t Know |
| Muscle tremors | ___ Yes ___ No ___ Don’t Know | ___ Yes ___ No ___ Don’t Know |
| Difficulty breathing | ___ Yes ___ No ___ Don’t Know | ___ Yes ___ No ___ Don’t Know |
| Fallen down suddenly | ___ Yes ___ No ___ Don’t Know | ___ Yes ___ No ___ Don’t Know |
| Swelling  *[If yes, select swollen body part]* | ___ Yes ___ No ___ Don’t Know | ___ Yes ___ No ___ Don’t Know |
|  | ___ Tongue ___ Throat ___ Sternum  ___ Flanks ___ Perineum  ___ Other:____________________ | ___ Tongue ___ Throat ___ Sternum  ___ Flanks ___ Perineum  ___ Other:___________________ |
| Drop in milk production | ___ Yes ___ No ___ Don’t Know | ___ Yes ___ No ___ Don’t Know |
| Discolored milk (Color____________) | ___ Yes ___ No ___ Don’t Know  ___ Not applicable | ___ Yes ___ No ___ Don’t Know  ___ Not applicable |
| Sudden death (within 8 hours of onset of clinical symptoms) | ___ Yes ___ No ___ Don’t Know | ___ Yes ___ No ___ Don’t Know |
| Found dead | ___ Yes ___ No ___ Don’t Know | ___ Yes ___ No ___ Don’t Know |
| Rapid bloating of the dead animal | ___ Yes ___ No ___ Don’t Know  ___ Not applicable | ___ Yes ___ No ___ Don’t Know  ___ Not applicable |
| Lack of stiffness of the body after death | ___ Yes ___ No ___ Don’t Know  ___ Not applicable | ___ Yes ___ No ___ Don’t Know  ___ Not applicable |
| Blood did not clot | ___ Yes ___ No ___ Don’t Know  ___ Not applicable | ___ Yes ___ No ___ Don’t Know  ___ Not applicable |
| Dark/Tarry colored blood observed | ___ Yes ___ No ___ Don’t Know  ___ Not applicable | ___ Yes ___ No ___ Don’t Know  ___ Not applicable |
| Bloody discharge from carcass | ___ Yes ___ No ___ Don’t Know  ___ Not applicable | ___ Yes ___ No ___ Don’t Know  ___ Not applicable |
| Swollen spleen of the dead animal | ___ Yes ___ No ___ Don’t Know  ___ Not applicable | ___ Yes ___ No ___ Don’t Know  ___ Not applicable |
| Other: Specify________________  ____________________________ | ___ Yes ___ No ___ Don’t Know | ___ Yes ___ No ___ Don’t Know |

**The next several questions are about the HERD, which is the entire group of animals that are pastured together.**

1. Were there scavenger or predator animals observed around the herd during PERIOD 1?

| Respondent 1 | Respondent 2 |
| --- | --- |
| ___ Yes  ___ No  ___ Don’t Know | ___ Yes  ___ No  ___ Don’t Know |

*[If No or Don’t Know, go to Question 38]*

- 1. If Yes, what animals did you see (indicate all that apply):

| Respondent 1 | Respondent 2 |
| --- | --- |
| ___ Wild pigs  ___ Stray dogs  ___ Wolves or Jackals  ___ Scavenging birds  ___ Other ____________________ | ___ Wild pigs  ___ Stray dogs  ___ Wolves or Jackals  ___ Scavenging birds  ___ Other ___________________ |

| Respondent 1 | Respondent 2 |
| --- | --- |
| ___ Yes  ___ No  ___ Don’t Know | ___ Yes  ___ No  ___ Don’t Know |

1. Did you notice more blood-sucking insects (horseflies) biting your animals than normal during PERIOD 1?

*[If No or Don’t Know, go to Question 39]*

- 1. If yes, specify insect if known

| Respondent 1 | Respondent 2 |
| --- | --- |
| ______________________ | _______________________ |

## Part 7. FEEDING PRACTICES

1. How were all of your [*same species as case*] animals fed during PERIOD 1?

| Respondent 1 | Respondent 2 |
| --- | --- |
| ___ Grazing only  ___ Grazing and trough feeding  ___ Trough feeding only | ___ Grazing only  ___ Grazing and trough feeding  ___ Trough feeding only |

*Go to Part 8*
39.1 Since your animals were fed from a trough, please tell me the kinds of food they were given DURING PERIOD ONE. [For each type of food given, ask where the food came from].

| Food Type | Was this type of food given? | Source of food | Where was it from? |
| --- | --- | --- | --- |
| Fresh cut green leaves/grasses/plants | ___ Yes  ___ No  ___ Don’t Know | □ From owner’s farm  □ From local farm  □ From store | Region ________________________  Municipality ___________________  Village _______________________  ___ Don’t Know |
| Roughages (dry plants such as hay, straw, maize) | ___ Yes  ___ No  ___ Don’t Know | □ From owner’s farm  □ From local farm  □ From store | Region ________________________  Municipality ___________________  Village _______________________  ___ Don’t Know |
| Concentrate | ___ Yes  ___ No  ___ Don’t Know | □ From owner’s farm  □ From local farm  □ From store | Region ________________________  Municipality ___________________  Village _______________________  ___ Don’t Know |
| Salt lick blocks | ___ Yes  ___ No  ___ Don’t Know | □ From owner’s farm  □ From local farm  □ From store | Region ________________________  Municipality ___________________  Village _______________________  ___ Don’t Know |
| Other:  __________ | ___ Yes  ___ No  ___ Don’t Know | □ From owner’s farm  □ From local farm  □ From store | Region ________________________  Municipality ___________________  Village _______________________  ___ Don’t Know |

*[If only fed from a trough, go to Part 9]*

## Part 8. PASTURE PRACTICES:

*[If “Grazing” or “Grazing and trough feeding” were selected in Question 39, complete this section]*

**I am going to ask about pasture feeding during PERIOD 1, which is the 30 days before DATE 1.**

1. What was the type of pasture or forage the herd grazed?

| Respondent 1 | Respondent 2 |
| --- | --- |
| ___ Mixed grasses  ___ Rye  ___ Alfalfa  ___ Other ______________________  ___ Don’t know | ___ Mixed grasses  ___ Rye  ___ Alfalfa  ___ Other ________________________  ___ Don’t know |

1. Were fertilizers used on the pasture on which the herdwas grazed ?

| Respondent 1 | Respondent 2 |
| --- | --- |
| ___ Yes  ___ No  ___ Don’t Know | ___ Yes  ___ No  ___ Don’t Know |

*[If No or Don’t Know, go to Question 42]*

- 1. If yes, please indicate the type of fertilizer(s):

| Respondent 1 | Respondent 2 |
| --- | --- |
| _____________________ | _______________________ |

1. Was there an animal anthrax burial site on or near the pastureland used by the herd?

| Respondent 1 | Respondent 2 |
| --- | --- |
| ___ Yes, on or adjacent to pasture  ___ Yes, within 1 km  ___ No  ___ Don’t Know | ___ Yes, on or adjacent to pasture  ___ Yes, within 1 km  ___ No  ___ Don’t Know |

1. Were there any earthworks or digging in the soil (such as plowing; gardening; ditch-digging; road, bridge or rail work; and irrigation channels) on or near to the pastureland used by the herd?

| Respondent 1 | Respondent 2 |
| --- | --- |
| ___ Yes, on or adjacent to pasture  ___ Yes, within 1 km  ___ No  ___ Don’t Know | ___ Yes, on or adjacent to pasture  ___ Yes, within 1 km  ___ No___ Don’t Know |

1. What was the condition of forage on the land on which your herd has been pastured in PERIOD 1?

| Respondent 1 | Respondent 2 |
| --- | --- |
| ___ Dry and rough  ___ Fresh and green  ___ Don’t Know | ___ Dry and rough  ___ Fresh and green  ___ Don’t Know |

**Part 9. WATER**

1. What is the source of water on the pastureland that your herd had access to during PERIOD 1? *[check all that apply]*

| Respondent 1 | Respondent 2 |
| --- | --- |
| ___ Pooling rainwater  ___ Pooling floodwater  ___ Permanent pond or lake  ___ Stream or creek  ___ River  ___ Piped water  ___ Other __________________ | ___ Pooling rainwater  ___ Pooling floodwater  ___ Permanent pond or lake  ___ Stream or creek  ___ River  ___ Piped water  ___ Other __________________ |

**Part 10. VETERINARY INTERVENTIONS**

1. Did your herd receive any veterinary care for any reason in the 6 months before DATE 1?

| Respondent 1 | Respondent 2 |  |
| --- | --- | --- |
| ___ Yes  ___ No  ___ Don’t Know | ___ Yes  ___ No  ___ Don’t Know | *[If No or Don’t know, go to 46.2]* |

- 1. If yes, from whom?

| Respondent 1 | Respondent 2 |
| --- | --- |
| ___ Government veterinarian  ___ Private veterinarian  ___ An animal caretaker who is a veterinarian/vet tech  ___ Don’t Know | ___ Government veterinarian  ___ Private veterinarian  ___ An animal caretaker who is a veterinarian/vet tech  ___ Don’t Know |

- - 1. For what reason? [check all that apply]

| Respondent 1 | Respondent 2 |
| --- | --- |
| ___ Vaccinate at my request  ___ Vaccinate as part of campaign  ___ Care for sick animal  ___ Assist with birthing  ___ Assist with accident/trauma  ___ General health check  ___ Other __________________  ___ Don’t Know | ___ Vaccinate at my request  ___ Vaccinate as part of campaign  ___ Care for sick animal  ___ Assist with birthing  ___ Assist with accident/trauma  ___ General health check  ___ Other __________________  ___ Don’t Know |

- 1. [*If 46 was no*] Why has a veterinarian not visited your herd in the 6 months before DATE 1?

| Respondent 1 | Respondent 2 |
| --- | --- |
| ___ Veterinary service is far away from household  ___ Veterinary service not available  ___ Do not have a financial possibility  ___ I treat my animals myself  ___ My neighbor treats my animals for me  ___ Do not think it is necessary or needed at all  ___ Animals did not need veterinary care  ___ Other (Specify:______________________) | ___ Veterinary service is far away from household  ___ Veterinary service not available  ___ Do not have a financial possibility  ___ I treat my animals myself  ___ My neighbor treats my animals for me  ___ Do not think it is necessary or needed at all  ___ Animals did not need veterinary care  ___ Other (Specify:______________________) |

1. Did veterinarians vaccinate any livestock in your village against anthrax in the 2 years before DATE 1?

| Respondent 1 | Respondent 2 |
| --- | --- |
| ___ Yes  ___ No  ___ Don’t Know/Don’t Remember | ___ Yes  ___ No  ___ Don’t Know/Don’t Remember |

[*Respondent 1: Yes, go to 47.1]*

[*Respondent 2: Yes, go to 48*]

- 1. *[Only ask Respondent 1]* How many of each animal species that you own received anthrax vaccine, and when were they vaccinated? How many of each animal species did you own at the time of vaccination?

| Animal | Select One | Number vaccinated | Date of most recent vaccinatation(mm/yy) | Number owned |
| --- | --- | --- | --- | --- |
| Cattle | ___ Yes ___ No ___ Don’t Know |  |  |  |
| Horse | ___ Yes ___ No ___ Don’t Know |  |  |  |
| Goat | ___ Yes ___ No ___ Don’t Know |  |  |  |
| Sheep | ___ Yes ___ No ___ Don’t Know |  |  |  |
| Pig | ___ Yes ___ No ___ Don’t Know |  |  |  |
| Other, specify:  __________________ | ___ Yes ___ No ___ Don’t Know |  |  |  |

1. Did you observe any side effects of your animals after anthrax vaccination?

| Respondent 1 | Respondent 2 |
| --- | --- |
| ___ Yes  ___ No  ___ Don’t Know | ___ Yes  ___ No  ___ Don’t Know |

- 1. If yes, describe the side effects you observed:

| Respondent 1 | Respondent 2 |
| --- | --- |
| _______________________________ | _______________________________ |

**Part 11. KAP**

**Now I will ask you your opinions of anthrax disease. Is it ok to proceed?**

1. Have you ever had anthrax or has a person you know ever had anthrax before PERIOD 1?

| Respondent 1 | Respondent 2 |
| --- | --- |
| ___ Yes  ___ No  ___ Don’t Know | ___ Yes  ___ No  ___ Don’t Know |

1. Have you or someone you know ever had an animal die from anthrax before PERIOD 1?

| Respondent 1 | Respondent 2 |
| --- | --- |
| ___ Yes  ___ No  ___ Don’t Know | ___ Yes  ___ No  ___ Don’t Know |

1. Have you received any informationon the identification or prevention of animal anthrax before PERIOD 1?

| Respondent 1 | Respondent 2 |
| --- | --- |
| ___ Yes  ___ No  ___ Don’t Know | ___ Yes  ___ No  ___ Don’t Know |

1. Have you received any information on the identification or prevention of animal anthrax after PERIOD 1?

| Respondent 1 | Respondent 2 |
| --- | --- |
| ___ Yes  ___ No  ___ Don’t Know | ___ Yes  ___ No  ___ Don’t Know |

1. How would you like to receive anthrax information from each of the following sources of If there is another source of information for anthrax please tell us

| Respondent 1 | Respondent 2 |
| --- | --- |
| ___ Veterinarian during vaccination campaigns  ___ Veterinarian when there is a health problem  ___ From agricultural consultant center  ___ From leaders at village meetings  ___ Listen to radio; TV  Other, specify: ____________________________ | ___ Veterinarian during vaccination campaigns  ___ Veterinarian when there is a health problem  ___ From agricultural consultant center  ___ From leaders at village meetings  ___ Listen to radio; TV  Other, specify: __________________________ |

1. I will read a list of animals, and tell me which ones you feel may be at risk for anthrax infection

| Respondent 1 | Respondent 2 |
| --- | --- |
| Y N Y N | Y N Y N |
| □ □ Cattle □ □ Sheep  □ □ Goat □ □ Pigs  □ □ Horse □ □ Dog  □ Others □ Don’t know | □ □ Cattle □ □ Sheep  □ □ Goat □ □ Pigs  □ □ Horse □ □ Dog  □ Others □ Don’t know |

1. Can people get anthrax from animals that have anthrax?

| Respondent 1 | Respondent 2 |
| --- | --- |
| ___ Yes  ___ No  ___ Don’t Know | ___ Yes  ___ No  ___ Don’t Know |

1. Can anthrax in animals be prevented?

| Respondent 1 | Respondent 2 |
| --- | --- |
| ___ Yes  ___ No  ___ Don’t Know | ___ Yes  ___ No  ___ Don’t Know |

1. Is anthrax a problem in your region?

| Respondent 1 | Respondent 2 |
| --- | --- |
| ___ Yes  ___ No  ___ Don’t Know | ___ Yes  ___ No  ___ Don’t Know |

[If No, go to 57.1]

- 1. Why do you think anthrax is NOT a problem?

| Respondent 1 | Respondent 2 |
| --- | --- |
| ______________________________________  ______________________________________ | __________________________________  ___________________________________ |

1. Would you vaccinate your animals for anthrax if the vaccine was free?

| Respondent 1 | Respondent 2 |
| --- | --- |
| ___ Yes  ___ No  ___ Don’t Know | ___ Yes  ___ No  ___ Don’t Know |

1. Would you vaccinate your animals for anthrax if you had to pay for the vaccine?

| Respondent 1 | Respondent 2 |
| --- | --- |
| ___ Yes  ___ No  ___ Don’t Know | ___ Yes  ___ No  ___ Don’t Know |

1. What do you do if one of your animals suddenly becomes sick *[check all that apply]*?

| Respondent 1 | Respondent 2 |
| --- | --- |
| ___ Treat it with antibiotics  ___ Slaughter it  ___ Separate it from the other animals  ___ Call a private veterinarian  ___ Call a government veterinarian  ___ Other: _______________________ | ___ Treat it with antibiotics  ___ Slaughter it  ___ Separate it from the other animals  ___ Call a private veterinarian  ___ Call a government veterinarian  ___ Other: _______________________ |

1. What do you do if one of your animals dies suddenly *[check all that apply]*?

| Respondent 1 | Respondent 2 |
| --- | --- |
| ___ Treat other animals with antibiotics  ___ Butcher it for meat  ___ Sell the carcass  ___ Bury the carcass  ___ Call a private veterinarian  ___ Call a government veterinarian  ___ Other: _______________________ | ___ Treat other animals with antibiotics  ___ Butcher it for meat  ___ Sell the carcass  ___ Bury the carcass  ___ Call a private veterinarian  ___ Call a government veterinarian  ___ Other: _______________________ |

1. In which languages would you prefer to receive educational materials on anthrax *[check all that apply]*?

| Respondent 1 | Respondent 2 |
| --- | --- |
| ___ Georgian  ___ Russian  ___ Azeri  ___ Armenian  ___ Other______________________ | ___ Georgian  ___ Russian  ___ Azeri  ___ Armenian  ___ Other_____________________ |

1. What was the highest level of education that you completed?

| Respondent 1 | Respondent 2 |
| --- | --- |
| ___ Primary  ___ Some of Secondary  ___ Secondary  ___ Certificate  ___ Some college  ___ College | ___ Primary  ___ Some of Secondary  ___ Secondary  ___ Certificate  ___ Some college  ___ College |

Additional comments during interview

____________________________________________________________________________________

____________________________________________________________________________________

____________________________________________________________________________________

Thank you for dedicating your time and participation to our survey, please accept this handout that shows ways to prevent anthrax in yourself, your family, and your animals. We wish you health and the best of luck!

Time interview ended: _____________________

*For Interviewer use only, to complete after the interview*

| Location | GPS coordinates |
| --- | --- |
| Location where animals are kept at night | 3 or more satellites? ___ Yes ___ No  N ____________ E_____________  Elevation__________ meters |

Notes on additional pasture history in past 30 days:__________________________________________________

___________________________________________________________________________________________

If more than 1 respondent was interviewed, were they interviewed at the same time?

___ Yes ___ No

How often were the respondents in agreement?

___ All the time ___ Some of the time ___ None of the time

**PART 12. ANIMAL CLASSIFICATION**

**Complete this section before travelling to the case’s village to conduct the interview**

Address where owner lives Name of Village/Town: _____________________________

Name of Municipality: ______________________________

Name of Region: ___________________________________

Date of disease onset (dd/mm/yy): __ __ / __ __ / __ __

If not available, Date of death (dd/mm/yy): __ __ / __ __ / __ __

Expertise number ____________________________ Expertise date (dd/mm/yyyy):___________________

Animal Identification number (if available): _________________________

Type of identification number: ___ Ear tag ___ Description of animal ___ Other, Specify___________

**Check the following boxes once the information has been collected**

| Check when complete | Tasks to complete before go to the field |
| --- | --- |
| ___ | The Questionnaire Identification Number has been provided by the project coordinators  Case Questionnaire Identification Number: __________ -_______- ___  Expertise number - year - code (1; vc1,2; ac1,2)  Village Control Questionnaire Identification Number: _______-______-___, _______-______-___  Area Control Questionnaire Identification Number: _______-______-___, _______-______-___ |
| ___ | You have written the Questionnaire Identificatoin Number on every page |
| ___ | You have to write Date1 (the date of disease onset for the case animal) on every page. If it is not available, please write the date of death. |
| ___ | Ensure the animal is a confirmed case, based on the below laboratory test(s) (select all that apply)  ___ Encapsulated bacilli on blood smear  ___ Positive bacterial culture  ___ PCR detection of virulence factor nucleic acid |
| ___ | Contact municipality vet and coordinate interviews |
| ___ | Check to see what language(s) the owner and shepherd speak, to determine which language version of the questionnaire to use, and if another interviewer is needed  ___ Georgian ___ Russian ___ Azerian ___ Armenian |
| ___ | The Area Control villages have been selected  Area control village 1: ___________________  Area control village 2: ___________________ |
| ___ | The animal species has been confirmed  Species ________________ |
