# Supplementary material for: Risk factors associated with the occurrence of anthrax outbreaks in livestock in the country of Georgia: A case-control investigation 2013-2015
Source: PLoS One. 2019 May 2;14(5):e0215228. doi: 10.1371/journal.pone.0215228 (PMC6497231; doi:10.1371/journal.pone.0215228)
Supplement: S2 Questionnaire — (DOCX) [file pone.0215228.s002.docx]

**ცხოველებში დაავადება ჯილეხის კვლევის კითხვარი**

**□ შემთხვევა □ სოფელი-კონტროლი □ არეალი-კონტროლი**

**ინტერვიუერის სახელი და გვარი: ______________________________________**

**ინტერვიუს აღების თარიღი** (დღე/თვე/წელი)**: __ __ /__ __ __ __ __ /__ __ __ __**

**ინტერვიუს დაწყების დრო: __ __ : __ __**

**შესავალი**

მე ვარ ……. [*სახელი, გვარი*] და ……. [*სახელები, გვარები*] არიან ჩემი კოლეგები. ჩვენ ვართ საქართველოს სოფლის მეურნეობის სამინისტროს სურსათის ეროვნული სააგენტოს წარმომადგენლები. მინდა გითხრათ, რომ საქართველოში სურსათის ეროვნული სააგენტოს მიერ “ცხოველთა ჯანმრთელობის ეროვნული პროგრამის” ფარგლებში ხორციელდება ცხოველებში დაავადება ჯილეხის კვლევა.

კონტროლი: ჩვენ გვსურს დაგისვათ რამდენიმე შეკითხვა თქვენი ....... [*იგივე სახეობა, რაც შემთხვევა*] შესახებ. გყავდათ თუ არა, ....... [*იგივე სახეობა, რაც შემთხვევა*] პერიოდი 1 განმავლობაში?

**[მკვლევარმა უნდა ჩაწეროს ცხოველი-შემთხვევის სახეობა და ყველა ცხოველი-კონტროლის სახეობა, იგევე უნდა იყოს რაც ცხოველი-შემთხვევის სახეობა]**

**[მკვლევარმა უნდა ახსნას, რომ პრეიოდი 1 ნიშნავს 30 დღეს თარიღი1-მდე]**

[თუ არ ჰყავდა პერიოდი 1 განმავლობაში ....... [*იგივე სახეობა, რაც შემთხვევა*]:

გმადლობთ, რომ გამოგვიყავით დრო, რადგან ჩვენ უნდა დავუსვათ შეკითხვები ცხოველთა იმ სახეობათა შესახებ, რომლებშიც გამოვლინდა ჯილეხის შემთხვევა. აღარ წაგართმევთ მეტ დროს. კარგ დღეს გისურვებთ.

შემთხვევა: კითხვები ეხება ....... [*იგივე სახეობა, რაც შემთხვევა*], რომლებშიც გამოვლინდა ჯილეხის შემთხვევა ....... [*თარიღი1*] და ასევე სხვა შინაურ ცხოველებს.

ინტერვიუ 30-45 წუთი გაგრძელდება. ის კონფიდენციალურია. მონაცემები გამოყენებული იქნება მხოლოდ აღნიშნული კვლევისათვის. ინტერვიუს პერიოდში ნებისმიერ დროს შეგვიძლია შევწყვიტოთ გამოკითხვა თქვენი სურვილისამებრ. გთხოვთ, გაითვალისწინოთ, რომ თქვენი მონაწილეობა დიდ წვლილს შეიტანს საქართველოში ჯილეხის ეფექტიანი პრევენციის საქმეში და გააუმჯობესებს აღნიშნული დაავადების კონტროლს.

**ხართ თანახმა მონაწილეობისათვის?**

**რესპოდენტი 1**  **რესპოდენტი 1**

□ დიახ □ არა □ დიახ □ არა

**რესპოდენტები:**

1. თქვენი სახელი? [***რესპოდენტი 1***]: ________________________________

თქვენ ხართ:

□ მეპატრონე □ მწყემსი/მომვლელი

1.1. ტელეფონის ნომერი: ________________________________

1. თქვენი სახელი? [***რესპოდენტი2***]: _________________________________

თქვენ ხართ:

□ მეპატრონე □ მეპატრონის ოჯახის წევრი □ მწყემსი/მომვლელი

2.1. ტელეფონის ნომერი: ________________________________

**ნაწილი1. ცხოველის მეპატრონე / მომვლელი**

**რესპოდენტი 1**

1. პერიოდი 1 განმავლობაში, 30 დღით ადრე ....... [*დაასახელეთ თარიღი 1 თარიღი*], ვინ უვლიდა თქვენს [*იგივე სახეობა, რაც ცხოველი]*?

□ მხოლოდ მე □ მე და ოჯახის წევრი (წევრები)

□ მხოლოდ ოჯახის წევრი(წევრები) □ მე, ოჯახის წევრები და მწყემსი

□ მე და მწყემსი □ მხოლოდ მწყემსი

[*თუ პასუხია “მხოლოდ მე”, გადადით მე-4 კითხვაზე*]

- 1. რადგან სხვებიც უვლიდნენ ცხოველს, შეუძლიათ მათ მონაწილეობა მიიღონ ინტერვიუში? ძალიან სასარგებლო იქნება, მათგანაც მოვისმინოთ პასუხები.

□ დიახ

□ არა

□ არ ვიცი

ვინაიდან არ შეგვიძლია გავესაუბროთ მათ, გვსურს გავიგოთ მათი სახელი, წვლილი და ტელეფონის ნომერი, რომ დავგეგმოთ მათთან ინტერვიუ.

3.2 სახელი ___________________________________

3.3 როლი/წვლილი ____________________________________

3.4 ტელეფონი___________________________________

1. საერთო ჯამში რამდენი ხანი უვლიდით ცხოველს პერიოდი 1 განმავლობაში?

□ მთელი პერიოდი □ დილას და საღამოს

□ დღის განმავლობაში □ ყოველ მეორე დღეს

□ კვირაში ერთხელ □ თვეში ერთხელ

□ საერთოდ არ ვუვლიდი პერიოდი1 განმავლობაში

4.1 რამდენ წლიანი გამოცდილება გაქვთ ცხოველების მოვლის? _______

1. რამდენი წლის ხართ? _______
2. სქესი:

□ მამრობითი □ მდედრობითი

**რესპოდენტი 2**

1. საერთო ჯამში რამდენი ხანი უვლიდით ცხოველს პერიოდი 1 განმავლობაში?

□ მთელი პერიოდი □ დილას და საღამოს

□ დღის განმავლობაში □ ყოველ მეორე დღეს

□ კვირაში ერთხელ □ თვეში ერთხელ

□ საერთოდ არ ვუვლიდი პერიოდი1 განმავლობაში

- 1. რამდენ წლიანი გამოცდილება გაქვთ ცხოველების მოვლის? _______

1. რამდენი წლის ხართ? _______
2. სქესი:

□ მამრობითი □ მდედრობითი

**ნაწილი2. მდგომარეობა და ისტორია**

შემთხვევა: მე ვაპირებ დაგისვათ შეკითხვა იმ ცხოველის შსახებ, რომელშიც გამოვლინდა ჯილეხი.

კონტროლი: მინდა გკითხოთ იმ ....... [*იგივე სახეობა, რაც შემთხვევა*] შესახებ, რომელსაც უვლიდით ბოლოს და რომელიც, ასევე, გყავდათ პერიოდი1-ს განმავლობაში.

1. ჰქონდა ცხოველს საყურე ნიშანი?

| რესპოდენტი1 | რესპოდენტი2 |
| --- | --- |
| □ დიახ  □ არა  □ არ ვიცი | □ დიახ  □ არა  □ არ ვიცი |

10.1

10.3

[*დადებითი პასუხის შემთხვევაში გადადით შეკითხვაზე 10.1, თუ პასუხია - “არა”, ან “არ ვიცი”, გადადით შეკითხვაზე 10.3*]

- 1. გახსოვთ ცხოველის ნომერი?

| რესპოდენტი1 | რესპოდენტი2 |
| --- | --- |
| □ დიახ  □ არა | □ დიახ  □ არა |

- 1. რა ნომერი იყო ცხოველის საყურე ნიშანი?

| რესპოდენტი1 | რესპოდენტი2 |
| --- | --- |
| ______________ | _______________ |

- 1. რა ერქვა და რა ფერის იყო ცხოველი?

| რესპოდენტი1 | რესპოდენტი2 |
| --- | --- |
| ______________ | _______________ |

1. ცხოველის წარმომავლობა?

| რესპოდენტი1 | რესპოდენტი2 |
| --- | --- |
| □ ადგილობრივი პირუტყვის ნამატი  □ მუნიციპალიტეტში ნაყიდი  □ ნაყიდი მუნიციპალიტეტის გარეთ  □ ნაყიდი ქვეყნის ფარგლებს გარეთ  □ სხვა __________________________ | □ ადგილობრივი პირუტყვის ნამატი  □ მუნიციპალიტეტში ნაყიდი  □ ნაყიდი მუნიციპალიტეტის გარეთ  □ ნაყიდი ქვეყნის ფარგლებს გარეთ  □ სხვა __________________________ |

1. ცხოველის სქესი:

| რესპოდენტი1 | რესპოდენტი 2 |
| --- | --- |
| □ მდედრობითი  □ მამრობითი | □ მდედრობითი  □ მამრობითი |

[*გადადით შეკითხვაზე 12.1*]

[*გადადით შეკითხვაზე12.2*]

- 1. [*თუ მდედრობითია*] თარიღი1 დროს ცხოველი იყო:

| რესპოდენტი 1 | რესპოდენტი2 |
| --- | --- |
| □ მაკე  □ მეწველი  □ არამეწველი  □ დეკეული | □ მაკე  □ მეწველი  □ არამეწველი  □ დეკეული |

- 1. [*თუ მამრობითია*] თარიღი 1 დროს, ცხოველი იყო:

| რესპოდენტი1 | რესპოდენტი 2 |
| --- | --- |
| □ არაკასტრირებული  □ კასტრირებული | □ არაკასტრირებული  □ კასტრირებული |

1. დაახლოებით რა ასაკის იყო ცხოველი თარიღი1 დროს?

| რესპოდენტი1 | რესპოდენტი2 |
| --- | --- |
| _____ □ წელი  _____ □ თვე  □ არ ვიცი | _____ □ წელი  _____ □ თვე  □ არ ვიცი |

1. რა მდგომარეობაში იყო ცხოველი თარიღი1 დროს?

| რესპოდენტი1 | რესპოდენტი 2 |
| --- | --- |
| □ გამხდარი  □ ნორმალური  □ მსუქანი  □ არ ვიცი | □ გამხდარი  □ ნორმალური  □ მსუქანი  □ არ ვიცი |

1. რამდენი ხანი იმყოფებოდა [*იგივე სახეობა, რაც შემთხვევა*] თქვენი ცხოველების ჯგუფში თარიღი 1 დროს?

| რესპოდენტი 1 | რესპოდენტი 2 |
| --- | --- |
| _____ □ წელი  _____ □ თვე  □ არ ვიცი | _____ □ წელი  _____ □ თვე  □ არ ვიცი |

1. ხომ არ შეგინიშნავთ ცხოველისათვის რაიმე ცვლილებები პერიოდი 1 მანძილზე?

| რესპოდენტი1 | რესპოდენტი2 |
| --- | --- |
| □ კი  □ არა  □ არ ვიცი | □ კი  □ არა  □ არ ვიცი |

[*თუ პასუხია - “არა”, ან “არ ვიცი, გადადით მე-17 შეკითხვაზე*]

- 1. დადებითი პასუხის შემთხვევაში, გთხოვთ აირჩიოთ, რა ცვლილებები შენიშნეთ:

| რესპოდენტი1 | რესპოდენტი2 |
| --- | --- |
| □ შემცირებული წველადობა  □ ნაყოფის მოგდება  □ 12 საათზე მეტხანს კვების შეწყვეტა  □ ცხელება (_____°C)  □ კოჭლობა  □ სუნთქვის გაძნელება  □ სხვა ____________________________ | □ შემცირებული წველადობა  □ ნაყოფის მოგდება  □ 12 საათზე მეტხანს კვების შეწყვეტა  □ ცხელება(_____°C)  □ კოჭლობა  □ სუნთქვის გაძნელება  □ სხვა ____________________________ |

1. აღინიშნებოდა თუ არა განსხვავება იმ ცხოველის კვებაში, რომელზეც ვსაუბრობთ, თქვენ სხვა ....... [*იგივე სახეობები, რაც შემთხვევა*] შედარებით პერიოდი 1 განმავლობაში?

| რესპოდენტი1 | რესპოდენტი 2 |
| --- | --- |
| □ დიახ | □ დიახ |
| □ არა  □ არ ვიცი  □ არ მყავს სხვა ცხოველები | □ არა  □ არ ვიცი  □ არ მყავს სხვა ცხოველები |
|  |  |

[*თუ პასუხია - “არა”, ან “არ ვიცი” გადადით მე-18 კითხვაზე* ]

[*თუ პასუხია - “არ მყავს სხვა ცხოველები” გადადით მე-19 კითხვაზე* ]

- 1. თუ პასუხი დადებითია, აღწერეთ

| რესპოდენტი 1 | რესპოდენტი 2 |
| --- | --- |
| ___________________________________ | ___________________________________ |

1. იყო თუ არა ის ცხოველი, რომელზეც ვსაუბრობთ, სხვა ....... [*იგივე სახეობები, რაც შემთხვევა*] შედარებით სხვაგვარად დაბინავებული პერიოდი 1 განმავლობაში?

| რესპოდენტი1 | რესპოდენტი2 |
| --- | --- |
| □ დიახ | □ დიახ |
| □ არა | □ არა |
| □ არ ვიცი | □ არ ვიცი |

[*თუ პასუხია “არა” ან “არ ვიცი” გადადით მე-19 შეკითხვაზე* ]

18.1თუ პასუხი დადებითია, აღწერეთ:

| რესპოდენტი 1 | რესპოდენტი 2 |
| --- | --- |
| ___________________________________ | ___________________________________ |

1. როდესმე თუ ყოფილა თქვენი ცხოველი აცრილი ჯილეხის წინააღმდეგ სიცოცხლის განმავლობაში?

| რესპოდენტი1 | რესპოდენტი2 |
| --- | --- |
| □ დიახ | □ დიახ |
| □ არა | □ არა |
| □ არ ვიცი | □ არ ვიცი |

[*თუ პასუხია - “არა”, ან “არ ვიცი” გადადით მე-20 შეკითხვაზე*]

- 1. თუ პასუხი დადებითია, რამდენჯერ იყო ცხოველი აცრილი ჯილეხის წინააღმდეგ?

| რესპოდენტი 1 | რესპოდენტი2 |
| --- | --- |
| __________________________________ | __________________________________ |

- 1. ბოლოს როდის აიცრა ცხოველი ჯილეხის წინააღმდეგ?

| რესპოდენტი1 | რესპოდენტი 2 |
| --- | --- |
| □  □ თარიღი1-მდე 1 თვეზე ნაკლები  □ 1-6 თვით ადრე თარიღი1-მდე  □ 6-12 თვით ადრე თარიღი 1-მდე  □ 12 თვეზე მეტი თარიღი1-მდე | □  □ თარიღი1-მდე 1 თვეზე ნაკლები  □ 1-6 თვით ადრე თარიღი1-მდე  □ 6-12 თვით ადრე თარიღი 1-მდე  □ 12 თვეზე მეტი თარიღი1-მდე |

1. იყო თუ არა ცხოველი აცრილი ჯილეხის გარდა სხვა დაავადების წინააღმდეგ ბოლო 6 თვის განმავლობაში თარიღი1-მდე?

| რესპოდენტი1 | რესპოდენტი2 |
| --- | --- |
| □ დიახ | □ დიახ |
| □ არა | □ არა |
| □ არ ვიცი/არ მახსოვს | □ არ ვიცი/არ მახსოვს |

[*თუ პასუხია - “არა”, ან “არ ვიცი” გადადით 21-ე შეკითხვაზე*]

- 1. თუ პასუხი დადებითია, აღნიშნეთ დაავადება და ვაქცინაციის ჩატარების თარიღი:

| რესპოდენტი 1 | | რესპოდენტი 2 | |
| --- | --- | --- | --- |
| დაავადებები | ბოლო ვაქცინაციის თარიღი (*თვე, წელი*) | დაავადებები | ბოლო ვაქცინაციის თარიღი (*თვე, წელი*) |
| □ თურქული |  | □ თურქული |  |
| □ პასტერელოზი |  | □ პასტერილოზი |  |
| □ ბრუცელოზი |  | □ რუცელოზი |  |
| □ ბრადზოტი |  | □ ბრადზოტი |  |
| □ სხვა _____________ |  | □ სხვა ______________ |  |

1. იყო თუ არა ცხოველი დამუშავებული ექტო და ენდო პარაზიტებზე ბოლო 6 თვის განმავლობაში თარიღი1-მდე?

| რესპოდენტი1 | რესპოდენტი2 |
| --- | --- |
| □ დიახ | □ დიახ |
| □ არა | □ არა |
| □ არ ვიცი/არ მახსოვს | □ არ ვიცი/არ მახსოვს |

[*თუ პასუხია - “არა”, ან “არ ვიცი” გადადით 22-ე შეკითხვაზე*]

- 1. დადებითი პასუხის შემთხვევაში, მიუთითეთ თარიღი:

| რესპოდენტი 1 | რესპოდენტი2 |
| --- | --- |
| __________________ | __________________ |

**ნაწილი3.** **კლინიკური ინფორმაცია [მხოლოდ შემთხვევის დროს]**

1. რა იყო ჯილეხის გამოსავალი? [*წაუკითხეთ თითოეული*]

| რესპოდენტი1 | რესპოდენტი2 |
| --- | --- |
| □ ავადობა  □ დაკვლა დაავადების გამოვლენისთანავე  □ დაკვლა დაავადების სიმპტომების  გამოვლენამდე (ჯილეხი დადასტურდა  ცხოველის დაკვლის შემდეგ)  □ დაცემული ცხოველის დაკვლა  □ გამოჯანმრთელება  □ სიკვდილი  □ გაყიდვა, საბოლოო შედეგი უცნობია  □ სხვა _____________________________ | □ ავადობა  □ დაკვლა დაავადების გამოვლენისთანავე  □ დაკვლა დაავადების სიმპტომების  გამოვლენამდე (ჯილეხი გამოვლინდა  ცხოველის დაკვლის შემდეგ)  □ დაცემული ცხოველის დაკვლა  □ გამოჯანმრთელება  □ სიკვდილი  □ გაყიდვა, საბოლოო შედეგი უცნობია  □ სხვა _____________________________ |

- 1. რა პერიოდი გაგრძელდა დაავადება დაწყებიდან მის საბოლოო შედეგამდე?

| რესპოდენტი1 | რესპოდენტი2 |
| --- | --- |
| □ იპოვეს დაცემული  □ ნახევარ დღეზე ნაკლები  □ ნახევარი დღიდან 24 სთ  □ 24 სთ-ზე მეტი | □ იპოვეს დაცემული  □ ნახევარ დღეზე ნაკლები  □ ნახევარი დღიდან 24 სთ  □ 24 სთ-ზე მეტი |

- 1. თუ დაავადება გაგრძელდა ერთ დღეზე მეტხანს, მიუთითეთ დღეების რაოდენობა.

| რესპოდენტი1 | რესპოდენტი2 |
| --- | --- |
| ________ | __________ |

1. ხომ არ შეგინიშნავთ ავადმყოფობის შემდეგი კლინიკური ნიშნები თქვენს ცხოველში მისი სიცოცხლის პერიოდში, ან დაცემის შემდეგ? სიმპტომების ჩამოთვლისას მიპასუხეთ - “**კი**”, “**არა**”, “**არ ვიცი**”.

[*კითხვებზე, რომლებიც განკუთვნილია უკვე დაცემული ცხოველებისათვის, ცოცხალი ცხოველის შემთხვევაში მონიშნეთ პასუხი - “არ ექვემდებარება შევსებას”*].

| **კლინიკური ნიშნები** | **რესპოდენტი1** | **რესპოდენტი2** |
| --- | --- | --- |
| ცხელება | □ დიახ □ არა □ არ ვიცი | □ დიახ □ არა □ არ ვიცი |
| კვების შეწყვეტა | □ დიახ □ არა □ არ ვიცი | □ დიახ □ არა □ არ ვიცი |
| დიარეა | □ დიახ □ არა □ არ ვიცი | □ დიახ □ არა □ არ ვიცი |
| კრუნჩხვა | □ დიახ □ არა □ არ ვიცი | □ დიახ □ არა □ არ ვიცი |
| კუნთების ტრემორი (კანკალი) | □ დიახ □ არა □ არ ვიცი | □ დიახ □ არა □ არ ვიცი |
| სუნთქვის გაძნელება | □ დიახ □ არა □ არ ვიცი | □ დიახ □ არა □ არ ვიცი |
| უეცარი დაცემა | □ დიახ □ არა □ არ ვიცი | □ დიახ □ არა □ არ ვიცი |
| ორგანოების შესიება  [*დადებითი პასუხის შემთხვევაში მონიშნეთ ორგანო*] | □ დიახ □ არა □ არ ვიცი | □ დიახ □ არა □ არ ვიცი |
|  | □ ენა □ ყელი □ მკერდის ძვალი  □ ფერდი □ შორისი  □ სხვა: ____________________ | □ ენა □ ყელი □ მკერდის ძვალი □ ფერდი □ შორისი  □ სხვა: ____________________ |
| წველადობის შემცირება | □ დიახ □ არა □ არ ვიცი  □ არ ექვემდებარება შევსებას | □ დიახ □ არა □ არ ვიცი  □ არ ექვემდებარება შევსებას |
| ფერშეცვლილი რძე (*მიუთითეთ ფერი* ___________ ) | □ დიახ □ არა □ არ ვიცი □ არ ექვემდებარება შევსებას | □ დიახ □ არა □ არ ვიცი  □ არ ექვემდებარება შევსებას |
| უეცარი დაცემა (კლინიკური ნიშნების გამოვლენიდან 8 საათის განმავლობაში) | □ დიახ □ არა □ არ ვიცი | □ დიახ □ არა □ არ ვიცი |
| დაცემულის პოვნა | □ დიახ □ არა □ არ ვიცი | □ დიახ □ არა □ არ ვიცი |
| ლეშის ძლიერი გაბერილობა | □ დიახ □ არა □ არ ვიცი  □ არ ექვემდებარება შევსებას | □ დიახ □ არა □ არ ვიცი  □ არ ექვემდებარება შევსებას |
| ლეშს არ აღენიშნება გაშეშება | □ დიახ □ არა □ არ ვიცი  □ არ ექვემდებარება შევსებას | □ დიახ □ არა □ არ ვიცი  □ არ ექვემდებარება შევსებას |
| სისხლის შეუდედებლობა | □ დიახ □ არა □ არ ვიცი  □ არ ექვემდებარება შევსებას | □ დიახ □ არა □ არ ვიცი  □ არ ექვემდებარება შევსებას |
| აღინიშნებოდა მუქი/ბლანტი სისხლი | □ დიახ □ არა □ არ ვიცი  □ არ ექვემდებარება შევსებას | □ დიახ □ არა □ არ ვიცი  □ არ ექვემდებარება შევსებას |
| სისხლიანი გამონადენი ბუნებრივი ხვრელებიდან | □ დიახ □ არა □ არ ვიცი  □ არ ექვემდებარება შევსებას | □ დიახ □ არა □ არ ვიცი  □ არ ექვემდებარება შევსებას |
| შესიებული ელენთა | □ დიახ □ არა □ არ ვიცი  □ არ ექვემდებარება შევსებას | □ დიახ □ არა □ არ ვიცი  □ არ ექვემდებარება შევსებას |
| სხვა: _____________________ | □ დიახ □ არა □ არ ვიცი | □ დიახ □ არა □ არ ვიცი |

**ნაწილი 4.** **ნახირის დემოგრაფია [*შემთხვევისა და კონტროლის შემთხვევაში*]**

მინდა გკითხოთ ცხოველთა იმ ჯგუფის შესახებ, რომელთანაც იმყოფებოდა თქვენი ცხოველი, რომელზეც ვსაუბრობთ პერიოდი 1 განმავლობაში. ეს მოიცავს საძოვრებზე ერთად ყოფნას, კვებას, გაადგილებას და შესაძლოა, ჰყავდეთ სხვა მფლობელები. ამ ჯგუფს ვუწოდებ ნახირს. გასაგებია ყველაფერი?

1. რამდენი ცხოველი იყო ნახირში სახეობათა მიხედვით პერიოდი 1 განმავლობაში? რამდენი მათგანი დაავადდა და რამდენი დაეცა? მონიშნეთ სახეობათა მიხედვით.

|  | რესპოდენტი1 | | | | რესპოდენტი 2 | | | |
| --- | --- | --- | --- | --- | --- | --- | --- | --- |
| სახეობები | ჯამი | ნორმალური | დაავადებული -ცოცხ. | დაცემული | ჯამი | ნორმალური | დაავადებული -ცოცხ. | დაცემული |
| მსხვილფეხა პირუტყვი |  |  |  |  |  |  |  |  |
| თხა |  |  |  |  |  |  |  |  |
| ცხვარი |  |  |  |  |  |  |  |  |
| ცხენი |  |  |  |  |  |  |  |  |
| ღორი |  |  |  |  |  |  |  |  |
| სხვა: ______________ |  |  |  |  |  |  |  |  |

- 1. რამდენი სხვა მეპატრონე ჰყავდა ზემოთ აღნიშნულ ცხოველებს?

| რესპოდენტი 1 | რესპოდენტი2 |
| --- | --- |
| □ 1  □ 2-25  □ 26-50  □ 51-100  □ 101-150  □ 151-200  □ >200 | □ 1  □ 2-25  □ 26-50  □ 51-100  □ 101-150  □ 151-200  □ >200 |

1. სად ინახავდით თქვენ ....... [იგივე სახეობები, რაც შემთხვევა] პერიოდი 1 განმავლობაში? [*მონიშნეთ ყველა შესაბამისი პასუხი*]

| რესპოდენტი1 | რესპოდენტი 2 |
| --- | --- |
| □ დახურული შემოფარგლული ტერიტორია  □ ადგილობრივი საძოვრები  □ შემოღობილი ტერიტორია  □ სეზონური საძოვრები  □ სხვა: ________________ | □ დახურული შემოფარგლული ტერიტორია  □ ადგილობრივი საძოვრები  □ შემოღობილი ტერიტორია  □ სეზონური საძოვრები  □ სხვა: ________________ |

- 1. თუ თქვენი ყველა ....... [იგივე სახეოები, რაც შემთხვევა] იმყოფებოდნენ ერთზე მეტ ადგილას, კვირის განმავლობაში რამდენჯერ გადაადგილდებოდნენ ერთი ადგილიდან მეორეზე პერიოდო 1 განმავლობაში?

| რესპოდენტი1 | რესპოდენტი2 |
| --- | --- |
| □ ყოველდღე  □ თითქმის ყოველდღე  □ რამდენიმე დღე  □ 1 დღე  □ არ ვიცი  □ არ გადაადგილდებოდნენ | □ ყოველდღე  □ თითქმის ყოველდღე  □ რამდენიმე დღე  □ 1 დღე  □ არ ვიცი  □ არ გადაადგილდებოდნენ |

[*თუ პასუხია - „არ გადაადგილდებოდნენ“, გადადით კითხვაზე 26*]

- 1. როგორ გადაადგილდებოდნენ ცხოველები ერთი ადგილიდან მეორეზე?

| რესპოდენტი1 | რესპოდენტი 2 |
| --- | --- |
| □ ფეხით  □ სატრანსპორტო საშუალებით | □ ფეხით  □ სატრანსპორტო საშუალებით |

- 1. განსხვავებულად გადაადგილდებოდა თუ არა სხვა ცხოველებთან შედარებით, ის ცხოველი, რომელზეც ვსაუბრობთ პერიოდი 1 განმავლობაში?

| რესპოდენტი1 | რესპოდენტი2 |
| --- | --- |
| □ დიახ  □ არა  □ არ ვიცი | □ დიახ  □ არა  □ არ ვიცი |

26. იყოფდა თუ არა ის ცხოველი [*იგივე სახეობა, რაც შემთხვევა*], რომელზეც ვსაუბრობთ, საკვებ ადგილს სხვა ცხოველებთან ერთად სოფელში?

| რესპოდენტი 1 | რესპოდენტი2 |
| --- | --- |
| □ კი | □ კი |
| □ არა | □ არა |
| □ არ ვიცი/არ მახსოვს | □ არ ვიცი/არ მახსოვს |
| □ არ ექვემდებარება შევსებას [არ გადაადგილდებოდნენ] | □ არ ექვემდებარება შევსებას  [არ გადაადგილდებოდნენ] |

[*გადადით 26.1-ე შეკითხვაზე*]

[*გადადით 27-ე შეკითხვაზე*]

26.1. დადებითი პასუხის შემთხვევაში, სად?

| რესპოდენტი 1 | რესპოდენტი 2 |
| --- | --- |
| □ საერთო საძოვრებზე  □ საერთო საკვებური/ბაგა  □ სხვა _________________ | □ საერთო საძოვრებზე  □ საერთო საკვებური/ბაგა  □ სხვა _________________ |

**ნაწილი 5**. **სეზონური მიგრაცია**

1. არის, თუ არა გადასარეკი ტრასა 1 კილომეტრის რადიუსში იმ საძოვართან, სადაც ძოვდა ნახირი პერიოდი1 განმავლობაში?

| რესპოდენტი1 | რესპოდენტი2 |
| --- | --- |
| □ დიახ  □ არა  □ არ ვიცი/არ მახსოვს | □ დიახ  □ არა  □ არ ვიცი/არ მახსოვს |

1. გადაადგილეთ თუ არა თქვენი ყველა ცხოველი [*იგივე სახეობები, რაც შემთხვევა*] სახლიდან სეზონურ საძოვარზე, ან პირიქით? (ზაფხულის, ან ზამთრის საძოვარი) პერიოდი 1 განმავლობაში?

| რესპოდენტი 1 | რესპოდენტი2 |
| --- | --- |
| □ დიახ, ყველა ცხოველი  □ დიახ, მაგრამ არა ყველა ცხოველი  □ არა  □ არ ვიცი | □ დიახ, ყველა ცხოველი  □ დიახ, მაგრამ არა ყველა ცხოველი  □ არა  □ არ ვიცი |

[გადადით 28.1.-ე შეკითხვაზე]

[გადადით 29-ე შეკითხვაზე]

- 1. როგორ გადაადგილდებოდნენ ისინი?

| რესპოდენტი1 | რესპოდენტი 2 |
| --- | --- |
| □ ფეხით  □ სატრანსპორტო საშუალებით | □ ფეხით  □ სატრანსპორტო საშუალები |

- 1. გადაადგილდებოდა, თუ არა ის ცხოველი, რომელზეც ვსაურობთ სხვა ცხოველებთან შედარებით განსხვავავებულად სეზონური საძოვრიდან, ან პირიქით?

| რესპოდენტი1 | რესპოდენტი 2 |
| --- | --- |
| □ დიახ | □ დიახ |
| □ არა | □ არა |
| □ არ ვიცი | □ არ ვიცი |

29. როდესმე თუ გამოგიყენებიათ სეზონური საძოვარი?

| რესპოდენტი1 | რესპოდენტი 2  [თუ პასუხია - „არა“, ან „არ ვიცი“, გადადით 35-ე კითხვაზე] |
| --- | --- |
| □ დიახ | □ დიახ |
| □ არა | □ არა |
| □ არ ვიცი/არ მახსოვს | □ არ ვიცი/არ მახსოვს |

30. სად მდებარეობს ზემოთ აღნიშნული სეზონური საძოვარი?

რეგიონი _______________________________

მუნიციპალიტეტი ___________________________

მდებარეობა ______________________________

საძოვარის დასახელება _____________________________

31. რომელ თვეში გადაგყავთ ცხოველები სახლიდან საძოვარზე?

| რესპოდენტი1 | რესპოდენტი 2 |
| --- | --- |
| □ თვე ___________________  □ იცვლება ყოველწლიურად | □ თვე ___________________  □ იცვლება ყოველწლიურად |
| □ არ ვიცი/არ მახსოვს | □ არ ვიცი/არ მახსოვს |

32. ბოლო გადაყვანისას რამდენი ხანი დაჭირდა თქვენი ცხოველების [*იგივე სახეობები, რაც შემთხვევა*] სეზონურ საძოვარზე გადაადგილებას? *(მიუთითეთ დღეების რაოდენობა*)

| რესპოდენტი 1 | რესპოდენტი2 |
| --- | --- |
| _________________  □ არ ვიცი/არ მახსოვს | _________________  □ არ ვიცი/არ მახსოვს |

33. რამდენი ხანი იმყოფებოდნენ თქვენი ცხოველები [იგივე სახეობები, რაც შემთხვევა] სეზონურ საძოვარზე ბოლო ჯერზე? (*მიუთითეთ დღეების რაოდენობა*)

| რესპოდენტი1 | რესპოდენტი2 |
| --- | --- |
| _________________  □ არ ვიცი/არ მახსოვს | _________________  □ არ ვიცი/არ მახსოვს |

34. რამდენი სული ცხოველი [*იგივე სახეობა, რაც შემთხვევა*] დაეცა ბოლოს საძოვრიდან, ან საძოვრისკენ გადაადგილების დროს ბოლო ჯერზე? (ასეთი ფაქტის შემთხვევაში)

| რესპოდენტი 1 | რესპოდენტი2 |
| --- | --- |
| _________________  □ არ ვიცი/არ მახსოვს | _________________  □ არ ვიცი/არ მახსოვს |

**ნაწილი 6.** მესაკუთრის ცხოველები

**მომდევნო რამდენიმე შეკითხვას დაგისვამთ მხოლოდ იმ ცხოველების შესახებ, რომლებიც თქვენ გყავდათ პერიოდი 1 განმავლობაში.**

35. [შემთხვევა]: გარდა ჯილეხით დაავადებული ხცოველისა, დაეცა თუ არა უეცრად თქვენი რომელიმე ცხოველი პერიოდი 1 განმავლობაში?

[კონტროლი]: დაეცა თუ არა თქვენი რომელიმე ცხოველი უეცრად პერიოდი 1 განმავლობაში?

| რესპოდენტი1 | რესპოდენტი2 |
| --- | --- |
| □ დიახ | □ დიახ |
| □ არა | □ არა |
| □ არ ვიცი/არ მახსოვს | □ არ ვიცი/არ მახსოვს |

[თუ პასუხია - “არა”, ან “არ ვიცი”, გადადით 36-ე შეკითხვაზე]

35.1. დადასტურდა, თუ არა, რომ ცხოველის დაცემის მიზეზი იყო ჯილეხი?

| რესპოდენტი1 | რესპოდენტი2 |
| --- | --- |
| □ დიახ  □ არა  □ არ ვიცი/არ მახსოვს | □ დიახ  □ არა  □ არ ვიცი/არ მახსოვს |

35.2. ვინ დაადგინა, რომ ცხოველის დაცემის მიზეზი იყო ჯილეხი? [*მონიშნეთ ყველა შესაბამისი პასუხი*]

| რესპოდენტი1 | რესპოდენტი2 |
| --- | --- |
| □ მფლობელი  □ ცხოველის მომვლელი  □ მეზობელი  □ ადგილობრივი ექიმი  □ კერძო ვეტერინარი  □ ლაბორატორია  □ სახელმწიფო ვეტერინარი  □ სხვა _________________ | □ მფლობელი  □ ცხოველის მომვლელი  □ მეზობელი  □ ადგილობრივი ექიმი  □ კერძო ვეტერინარი  □ ლაბორატორია  □ სახელმწიფო ვეტერინარი  □ სხვა _________________ |

35.3. რა ქმედებები განახორციელეთ? [*მონიშნეთ ყველა შესაბამისი*]

| რესპოდენტი1 | რესპოდენტი2 |
| --- | --- |
| □ ჯანმრთელი და ავადმყოფი ცხოველების განცალკევება  □ ანტიბიოტიკებით მკურნალობა  □ ჩაუტარდათ ჯილეხის საწინააღმდეგო  ვაქცინაცია  □ სადგომის დეზინფექცია  □ არაფერი | □ ჯანმრთელი და ავადმყოფი ცხოველების განცალკევება  □ ანტიბიოტიკებით მკურნალობა  □ ჩაუტარდათ ჯილეხის საწინააღმდეგო  ვაქცინაცია  □ სადგომის დეზინფექცია  □ არაფერი |

35.4. ხომ არ შეგინიშნავთ დაცემულ ცხოველში შემდეგი? კლინიკური ნიშნების ჩამოთვლისას მიპასუხეთ - “კი”, “არა”, ან “არ ვიცი”.

| **კლინიკური ნიშნები** | **რესპოდენტი1** | **რესპოდენტი2** |
| --- | --- | --- |
| ცხელება | □ დიახ □ არა □ არ ვიცი | □ დიახ □ არა □ არ ვიცი |
| კვების შეწყვეტა | □ დიახ □ არა □ არ ვიცი | □ დიახ □ არა □ არ ვიცი |
| დიარეა | □ დიახ □ არა □ არ ვიცი | □ დიახ □ არა □ არ ვიცი |
| კრუნჩხვა | □ დიახ □ არა □ არ ვიცი | □ დიახ □ არა □ არ ვიცი |
| კუნთების ტრემორი (კანკალი) | □ დიახ □ არა □ არ ვიცი | □ დიახ □ არა □ არ ვიცი |
| სუნთქვის გაძნელება | □ დიახ □ არა □ არ ვიცი | □ დიახ □ არა □ არ ვიცი |
| უეცარი დაცემა | □ დიახ □ არა □ არ ვიცი | □ დიახ □ არა □ არ ვიცი |
| ორგანოების შესიება  [*დადებითი პასუხის შემთხვევაში მონიშნეთ ორგანო*] | □ დიახ □ არა □ არ ვიცი | □ დიახ □ არა □ არ ვიცი |
|  | □ ენა □ ყელი □ მკერდის ძვალი  □ ფერდი □ შორისი  □ სხვა: ____________________ | □ ენა □ ყელი □ მკერდის ძვალი □ ფერდი □ შორისი  □ სხვა: ____________________ |
| წველადობის შემცირება | □ დიახ □ არა □ არ ვიცი  □ არ ექვემდებარება შევსებას | □ დიახ □ არა □ არ ვიცი  □ არ ექვემდებარება შევსებას |
| ფერშეცვლილი რძე (*მიუთითეთ ფერი* ___________ ) | □ დიახ □ არა □ არ ვიცი □ არ ექვემდებარება შევსებას | □ დიახ □ არა □ არ ვიცი  □ არ ექვემდებარება შევსებას |
| უეცარი დაცემა (კლინიკური ნიშნების გამოვლენიდან 8 საათის განმავლობაში) | □ დიახ □ არა □ არ ვიცი | □ დიახ □ არა □ არ ვიცი |
| დაცემულის პოვნა | □ დიახ □ არა □ არ ვიცი | □ დიახ □ არა □ არ ვიცი |
| ლეშის ძლიერი გაბერილობა | □ დიახ □ არა □ არ ვიცი  □ არ ექვემდებარება შევსებას | □ დიახ □ არა □ არ ვიცი  □ არ ექვემდებარება შევსებას |
| ლეშს არ აღენიშნება გაშეშება | □ დიახ □ არა □ არ ვიცი  □ არ ექვემდებარება შევსებას | □ დიახ □ არა □ არ ვიცი  □ არ ექვემდებარება შევსებას |
| სისხლის შეუდედებლობა | □ დიახ □ არა □ არ ვიცი  □ არ ექვემდებარება შევსებას | □ დიახ □ არა □ არ ვიცი  □ არ ექვემდებარება შევსებას |
| აღინიშნებოდა მუქი/ბლანტი სისხლი | □ დიახ □ არა □ არ ვიცი  □ არ ექვემდებარება შევსებას | □ დიახ □ არა □ არ ვიცი  □ არ ექვემდებარება შევსებას |
| სისხლიანი გამონადენი ბუნებრივი ხვრელებიდან | □ დიახ □ არა □ არ ვიცი  □ არ ექვემდებარება შევსებას | □ დიახ □ არა □ არ ვიცი  □ არ ექვემდებარება შევსებას |
| შესიებული ელენთა | □ დიახ □ არა □ არ ვიცი  □ არ ექვემდებარება შევსებას | □ დიახ □ არა □ არ ვიცი  □ არ ექვემდებარება შევსებას |
| სხვა: _____________________ | □ დიახ □ არა □ არ ვიცი | □ დიახ □ არა □ არ ვიცი |

**ნაწილი 7.** **ნახირის ცხოველები**

**ახლა შეგეკითხოთ სხვისი ცხოველების შესახებ, რომლებიც პეროდი 1 განმავლობაში ძოვდნენ თქვენს ცხოველებთან ერთად, მაგრამ ჰყავდათ სხვა მფლობელი.**

36. ხომ არ დაცემულა უეცრად რომელიმე ასეთი ცხოველი პერიოდი 1-ის განმავლობაში?

| რესპოდენტი 1 | რესპოდენტი 2 |
| --- | --- |
| □ დიახ  □ არა  □ არ ვიცი  □ არ ექვემდებარება შევსებას | □ დიახ  □ არა  □ არ ვიცი  □ არ ექვემდებარება შევსებას |

*[გადადით 37-ე შეკითხვაზე*]

36.1 დადასტურდა, თუ არა რომ ცხოველის დაცემის მიზეზი იყო ჯილეხი?

| რესპოდენტი 1 | რესპოდენტი 2 |
| --- | --- |
| □ დიახ  □ არა  □ არ ვიცი | □ დიახ  □ არა  □ არ ვიცი |

36.2. რა ქმედებები განახორციელეთ თქვენს ცხოველთან მიმართებაში?

| რესპოდენტი 1 | რესპოდენტი 2 |
| --- | --- |
| □ ჯანმრთელი და ავადმყოფი ცხოველების განცალკევება  □ ანტიბიოტიკებით მკურნალობა  □ ჩაუტარდათ ჯილეხის საწინააღმდეგო  ვაქცინაცია  □ სადგომის დეზინფექცია  □ არაფერი | □ ჯანმრთელი და ავადმყოფი ცხოველების განცალკევება  □ ანტიბიოტიკებით მკურნალობა  □ ჩაუტარდათ ჯილეხის საწინააღმდეგო  ვაქცინაცია  □ სადგომის დეზინფექცია  □ არაფერი |

36.3. დაცემულ ცხოველებში, ხომ არ შეგინიშნავთ შემდეგი კლინიკური ნიშნები? კლინიკური ნიშნების წაკითხვისას მიპასუხეთ - “დიახ”, “არა”, ან “არ ვიცი”.

| **კლინიკური ნიშნები** | **რესპოდენტი1** | **რესპოდენტი2** |
| --- | --- | --- |
| ცხელება | □ დიახ □ არა □ არ ვიცი | □ დიახ □ არა □ არ ვიცი |
| კვების შეწყვეტა | □ დიახ □ არა □ არ ვიცი | □ დიახ □ არა □ არ ვიცი |
| დიარეა | □ დიახ □ არა □ არ ვიცი | □ დიახ □ არა □ არ ვიცი |
| კრუნჩხვა | □ დიახ □ არა □ არ ვიცი | □ დიახ □ არა □ არ ვიცი |
| კუნთების ტრემორი (კანკალი) | □ დიახ □ არა □ არ ვიცი | □ დიახ □ არა □ არ ვიცი |
| სუნთქვის გაძნელება | □ დიახ □ არა □ არ ვიცი | □ დიახ □ არა □ არ ვიცი |
| უეცარი დაცემა | □ დიახ □ არა □ არ ვიცი | □ დიახ □ არა □ არ ვიცი |
| ორგანოების შესიება  [*დადებითი პასუხის შემთხვევაში მონიშნეთ ორგანო*] | □ დიახ □ არა □ არ ვიცი | □ დიახ □ არა □ არ ვიცი |
|  | □ ენა □ ყელი □ მკერდის ძვალი  □ ფერდი □ შორისი  □ სხვა: ____________________ | □ ენა □ ყელი □ მკერდის ძვალი □ ფერდი □ შორისი  □ სხვა: ____________________ |
| წველადობის შემცირება | □ დიახ □ არა □ არ ვიცი  □ არ ექვემდებარება შევსებას | □ დიახ □ არა □ არ ვიცი  □ არ ექვემდებარება შევსებას |
| ფერშეცვლილი რძე(*მიუთითეთ ფერი* __________) | □ დიახ □ არა □ არ ვიცი □ არ ექვემდებარება შევსებას | □ დიახ □ არა □ არ ვიცი  □ არ ექვემდებარება შევსებას |
| უეცარი დაცემა (კლინიკური ნიშნების გამოვლენიდან 8 საათის განმავლობაში) | □ დიახ □ არა □ არ ვიცი | □ დიახ □ არა □ არ ვიცი |
| დაცემულის პოვნა | □ დიახ □ არა □ არ ვიცი | □ დიახ □ არა □ არ ვიცი |
| ლეშის ძლიერი გაბერილობა | □ დიახ □ არა □ არ ვიცი  □ არ ექვემდებარება შევსებას | □ დიახ □ არა □ არ ვიცი  □ არ ექვემდებარება შევსებას |
| ლეშს არ აღენიშნება გაშეშება | □ დიახ □ არა □ არ ვიცი  □ არ ექვემდებარება შევსებას | □ დიახ □ არა □ არ ვიცი  □ არ ექვემდებარება შევსებას |
| სისხლის შეუდედებლობა | □ დიახ □ არა □ არ ვიცი  □ არ ექვემდებარება შევსებას | □ დიახ □ არა □ არ ვიცი  □ არ ექვემდებარება შევსებას |
| აღინიშნებოდა მუქი/ბლანტი სისხლი | □ დიახ □ არა □ არ ვიცი  □ არ ექვემდებარება შევსებას | □ დიახ □ არა □ არ ვიცი  □ არ ექვემდებარება შევსებას |
| სისხლიანი გამონადენი ბუნებრივი ხვრელებიდან | □ დიახ □ არა □ არ ვიცი  □ არ ექვემდებარება შევსებას | □ დიახ □ არა □ არ ვიცი  □ არ ექვემდებარება შევსებას |
| შესიებული ელენთა | □ დიახ □ არა □ არ ვიცი  □ არ ექვემდებარება შევსებას | □ დიახ □ არა □ არ ვიცი  □ არ ექვემდებარება შევსებას |
| სხვა: _____________________ | □ დიახ □ არა □ არ ვიცი | □ დიახ □ არა □ არ ვიცი |

შემდეგი რამდენიმე კითხვა ეხება ნახირს, ანუ ცხოველთა ჯგუფს, რომელიც ერთად ძოვს.

37. შეინიშნებოდა თუ არა ლეშისმჭამელი ან მტაცებელი ცხოველები ნახირთან ახლოს პერიოდი 1 განმავლობაში?

| რესპოდენტი 1 | რესპოდენტი 2 |
| --- | --- |
| □ დიახ  □ არა  □ არ ვიცი | □ დიახ  □ არა  □ არ ვიცი |

[*თუ პასუხია -“არა”, ან “არ ვიცი”, გადადით 38-ე შეკითხვაზე*]

37.1. დადებითი პასუხის შემთხვევაში, რომელი ცხოველი გინახავთ? [*მიუთითეთ ყველა საჭირო*]

| რესპოდენტი 1 | რესპოდენტი 2 |
| --- | --- |
| □ გარეული ტახი  □ უპატრონო ძაღლები  □ მგელი, ან ტურა  □ ლეშისმჭამელი ფრინველი  □ სხვა ____________________ | □ გარეული ტახი  □ უპატრონო ძაღლები  □ მგელი, ან ტურა  □ ლეშისმჭამელი ფრინველი  □ სხვა ____________________ |

38. პერიოდი 1-ის მანძილზე ხომ არ შეგინიშნავთ ჩვეულებრივზე მეტი სისხლისმწოველი მწერები, რომლებიც კბენდნენ თქვენს ცხოველებს?

| რესპოდენტი 1 | რესპოდენტი 2 |
| --- | --- |
| □ დიახ  □ არა  □ არ ვიცი | □ დიახ  □ არა  □ არ ვიცი |

**{ თუ პასუხია “არა” ან “არ ვიცი”, გადადით 39-ე შეკითხვაზე}**

38.1 დადებითი პასუხის შემთხვევაში დაასახელეთ მწერი (თუ იცით)

| რესპოდენტი 1 | რესპოდენტი 2 |
| --- | --- |
| _____________________ | ______________________ |

## ნაწილი 8. კვების პრაქტიკა

## 39. პერიოდი 1-ის მანძილზე როგორ იკვებებოდნენ თქვენი [*იგივე სახეობა, რაც შემთხვევა*] ცხოველები?

| რესპოდენტი 1 | რესპოდენტი 2 |
| --- | --- |
| □ მხოლოდ ძოვდა (*არჩევის შემთხვევაში*  *გადადით მე-8 ნაწილზე*)  □ ძოვება და ბაგური კვება  □ მხოლოდ ბაგური კვება | □ მხოლოდ ძოვდა (*არჩევის შემთხვევაში*  *გადადით მე-8 ნაწილზე*)  □ ძოვება და ბაგური კვება  □ მხოლოდ ბაგური კვება |

39.1თუ თქვენი ცხოველები იკვებებოდნენ ბაგური კვებით, გთხოვთ მიუთითოთ თუ რა სახის საკვებს იღებდნენ პერიოდო 1 განმავლობაში. (თითოეული საკვების სახეობას მიუთითეთ წყარო).

| **საკვების სახეობა** | **თითოეული სახეობისთვის** | **საკვების წყარო** | **წარმომავლობა** |
| --- | --- | --- | --- |
| მწვანე ფოთლები/ბალახი/მცენარეები | □ დიახ  □ არა  □ არ ვიცი | □ საკუთარი  □ სხავ ლოკალური ფერმიდან  □ შემოტანილი | რეგიონი _____________________ მუნიციპალიტეტი _____________  სოფელი _____________________  □ არ ვიცი |
| უხეში საკვები (თივა, ჩალა, სიმინდი) | □ დიახ  □ არა  □ არ ვიცი | □ საკუთარი  □ სხავ ლოკალური ფერმიდან  □ შემოტანილი | რეგიონი _____________________ მუნიციპალიტეტი _____________  სოფელი _____________________  □ არ ვიცი |
| კონცენტრატები | □ დიახ  □ არა  □ არ ვიცი | □ საკუთარი  □ სხავ ლოკალური ფერმიდან  □ შემოტანილი | რეგიონი _____________________ მუნიციპალიტეტი _____________  სოფელი _____________________  □ არ ვიცი |
| ქვამარილი | □ დიახ  □ არა  □ არ ვიცი | □ საკუთარი  □ სხავ ლოკალური ფერმიდან  □ შემოტანილი | რეგიონი _____________________ მუნიციპალიტეტი _____________  სოფელი _____________________  □ არ ვიცი |
| სხვა:______ | □ დიახ  □ არა  □ არ ვიცი | □ საკუთარი  □ სხავ ლოკალური ფერმიდან  □ შემოტანილი | რეგიონი _____________________ მუნიციპალიტეტი _____________  სოფელი _____________________  □ არ ვიცი |

[*მხოლოდ ბაგური კვების შემთხვევაში გადადით მე-9 ნაწილზე*]

## ნაწილი 9. ძოვების პრაქტიკა

[*თუ 39-ე კითხვაში მონიშნული იქნა - “ძოვება”, ან “ძოვება და ბაგური კვება” შეავსეთ ეს ნაწილი*]

მე შეგეკითხებით საძოვარზე კვების შესახებ პერიოდი 1 განმავლობაში.

40. რა სახის იყო საძოვარი ან ფორაჟი რომელზეც ძოვდა ნახირი?

| რესპოდენტი 1 | რესპოდენტი 2 |
| --- | --- |
| □ შერეული ბალახის  □ ჭვავის  □ იონჯას  □ სხვა ___________________  □ არ ვიცი | □ შერეული ბალახის  □ ჭვავის  □ იონჯას  □ სხვა ___________________  □ არ ვიცი |

41. იყენებდნენ თუ არა სასუქებს იმ საძოვარზე სადაც ძოვდა ნახირი?

| რესპოდენტი 1 | რესპოდენტი 2 |
| --- | --- |
| □ დიახ  □ არა  □ არ ვიცი | □ დიახ  □ არა  □ არ ვიცი |

[*თუ პასუხია - “არა”, ან “არ ვიცი”, გადადით 42-ე შეკითხვაზე*]

41.1. დადებითი პასუხის შემთხვევაში, გთხოვთ მიუთითოთ სასუქის სახეობები:

| რესპოდენტი 1 | რესპოდენტი 2 |
| --- | --- |
| _____________________ | ______________________ |

42. იყო, თუ არა ჯილეხით დაცემული ცხოველის სამარხი ნახირის მიერ გამოყენებულ საძოვარზე, ან მის ახლოს?

| რესპოდენტი 1 | რესპოდენტი 2 |
| --- | --- |
| □ დიახ საძოვარზე, ან მის ახლოს  □ დიახ 1კმ რადიუსში  □ არა  □ არ ვიცი | □ დიახ საძოვარზე, ან მის ახლოს  □ დიახ 1კმ რადიუსში  □ არა  □ არ ვიცი |

43. ხომ არ ჩატარებულა ნახირის მიერ გამოყენებულ საძოვარზე მიწის სამუშაოები, როგორიცაა: ხვნა, თესვა, არხის გაყვანა, ხიდის, გზის და რკინიგზის მშენებლობა?

| რესპოდენტი 1 | რესპოდენტი 2 |
| --- | --- |
| □ დიახ საძოვარზე, ან მის ახლოს  □ დიახ 1კმ რადიუსში  □ არა  □ არ ვიცი | □ დიახ საძოვარზე, ან მის ახლოს  □ დიახ 1კმ რადიუსში  □ არა  □ არ ვიცი |

44. რა მდგომარეობაში იყო ბალახი საძოვარზე, სადაც ძოვდა ნახირი პერიოდი 1 განმავლობაში?

| რესპოდენტი 1 | რესპოდენტი 2 |
| --- | --- |
| □ მშრალი და უხეში  □ ნორჩი და მწვანე  □ არ ვიცი | □ მშრალი და უხეში  □ ნორჩი და მწვანე  □ არ ვიცი |

**ნაწილი 10**. **წყალი**

45. როგორი წყლით ხდებოდა ნახირის დაწყურება საძოვარზე პერიოდი 1 განმავლობაში? [*მონიშნეთ ყველა შესაბამისი პასუხი*]

| რესპოდენტი 1 | რესპოდენტი 2 |
| --- | --- |
| □ დაგუბებული წვიმის წყალი  □ წყალდიდობის შედეგად  დაგუბებული წყალი  □ წყალსაცავი, ან ტბა  □ ნაკადული, ან პატარა გუბე  □ მდინარე  □ მილის წყალი  □ სხვა _________________ | □ დაგუბებული წვიმის წყალი  □ წყალდიდობის შედეგად  დაგუბებული წყალი  □ წყალსაცავი, ან ტბა  □ ნაკადული, ან პატარა გუბე  □ მდინარე  □ მილის წყალი  □ სხვა _________________ |

**ნაწილი 11.** **ვეტერინარული ჩარევა**

46. ხომ არ მიგიღიათ რაიმე ვეტერინარული მომსახურება თქვენი ნახირისთვის რაიმე მიზეზის გამო თარიღი1-მდე 6 თვით ადრე?

| რესპოდენტი 1 | რესპოდენტი 2 |  |
| --- | --- | --- |
| □ დიახ  □ არა  □ არ ვიცი | □ დიახ  □ არა  □ არ ვიცი | [*თუ პასუხია -“არა”, ან “არ ვიცი”, გადადით 46.2-ე შეკითხვაზე*] |

46.1. დადებითი პასუხის შემთხვევაში მიუთითეთ რომელი ვეტერინარის მომსახურებით ირასგებლეთ?

| რესპოდენტი 1 | რესპოდენტი 2 |
| --- | --- |
| □ სახელმწიფო ვეტერინარი  □ კერძო ვეტერინარი  □ ცხოველის მომვლელი ვინც არის ვეტერინარი ან ფერშალი  □ არ ვიცი | □ სახელმწიფო ვეტერინარი  □ კერძო ვეტერინარი  □ ცხოველის მომვლელი ვინც არის ვეტერინარი ან ფერშალი  □ არ ვიცი |

46.1.1. რა მიზეზით? [*მონიშნეთ ყველა საჭირო ვარიანტი*]

| რესპოდენტი 1 | რესპოდენტი 2 |
| --- | --- |
| □ ვაქცინაცია ჩემი მოთხოვნით  □ ვაქცინაცია, როგორც კამპანიის ნაწილი  □ მზრუნველობა დაავადებულ ცხოველზე  □ დახმარება მშობიარობის პროცესში  □ დახმარება უბედური  შემთხვევის/ტრავმის დროს  □ ჯამნრთელობის ზოგადი შემოწმება  □ სხვა _______________  □ არ ვიცი | □ ვაქცინაცია ჩემი მოთხოვნით  □ ვაქცინაცია, როგორც კამპანიის ნაწილი  □ მზრუნველობა დაავადებულ ცხოველზე  □ დახმარება მშობიარობის პროცესში  □ დახმარება უბედური  შემთხვევის/ტრავმის დროს  □ ჯამნრთელობის ზოგადი შემოწმება  □ სხვა _______________  □ არ ვიცი |

46.2. [იმ შემთხვევაში თუ 46-ე კითხვაზე იყო უარყოფითი პასუხი] რატომ არ მოინახულა ვეტერინარმა თქვენი ნახირი თარიღი 1-მდე 6 თვით ადრე?

| რესპოდენტი 1 | რესპოდენტი 2 |
| --- | --- |
| □ ვეტერინარული მომსახურება მეურნეობიდან  შორს მდებარეობს  □ ვეტერინარული მომსახურება არ არის  ხელმისაწვდომი  □ არ მაქვს ფინანსური შესაძლებლობა  □ მე თვითონ ვმკურნალობ საკუთარ ცხოველს  □ ჩემი მეზობელი მკურნალობს ჩემს ცხოველს  □ არ ვფიქრობ რომ ეს საჭირო ან აუცილებელია  □ არ დამჭირვებია  □ სხვა ___________________________ | □ ვეტერინარული მომსახურება მეურნეობიდან  შორს მდებარეობს  □ ვეტერინარული მომსახურება არ არის  ხელმისაწვდომი  □ არ მაქვს ფინანსური შესაძლებლობა  □ მე თვითონ ვმკურნალობ საკუთარ ცხოველს  □ ჩემი მეზობელი მკურნალობს ჩემს ცხოველს  □ არ ვფიქრობ რომ ეს საჭირო ან აუცილებელია  □ არ დამჭირვებია  □ სხვა ___________________________ |

47. ვეტერინარის მიერ თქვენს სოფელში აიცრა თუ არა პირუტყვი ჯილეხის წინააღმდეგ თარიღი 1-მდე 2 წლის განმავლობაში?

| რესპოდენტი 1 | რესპოდენტი 2 |
| --- | --- |
| □ დიახ  □ არა  □ არ ვიცი/არ მახსოვს | □ დიახ  □ არა  □ არ ვიცი/არ მახსოვს |

[*რესპოდენტი 1: დიახ გადადით 47.1-ე შეკითხვაზე*]

[*რესპოდენტი 2: დიახ გადადით 48-ე შეკითხვაზე*]

47.1. [*შეკითხვა მხოლოდ რესპოდენტი1-ს*] თქვენი რამდენი ცხოველი აიცრა ჯილეხის წინააღმდეგ და როდის სახეობების მიხედვით. რამდენი ცხოველი გყავდათ ვაქცინაციის პერიოდში?

| ცხოველი | მონიშნეთ ერთი პასუხი | აცრილი ცხოველების რაოდენობა | ბოლო აცრის თარიღი (თვე/წელი) | რამდენი ცხოველი გყავდათ |
| --- | --- | --- | --- | --- |
| მსხვილფეხა პირუტყვი | □ დიახ  □ არა  □ არ ვიცი |  |  |  |
| ცხენი | □ დიახ  □ არა  □ არ ვიცი |  |  |  |
| თხა | □ დიახ  □ არა  □ არ ვიცი |  |  |  |
| ცხვარი | □ დიახ  □ არა  □ არ ვიცი |  |  |  |
| ღორი | □ დიახ  □ არა  □ არ ვიცი |  |  |  |
| სხვა, დააკონკრეტეთ:  __________________ | □ დიახ  □ არა  □ არ ვიცი |  |  |  |

48. ხომ არ შეგინიშნავთ რაიმე გვერდითი მოვლენები ჯილეხის ვაქცინაციის შემდეგ?

| რესპოდენტი 1 | რესპოდენტი 2 |
| --- | --- |
| □ დიახ  □ არა  □ არ ვიცი | □ დიახ  □ არა  □ არ ვიცი |

48.1 დადებითი პასუხის შემთხვევაში აღწერეთ შენიშნული გვერდითი მოვლენები:

| რესპოდენტი 1 | რესპოდენტი 2 |
| --- | --- |
| _______________________________ | _____________________________ |

**ნაწილი 12.**

ახლა მაინტერესებს თქვენი აზრი დაავადება ჯილეხის შესახებ. გავაგრძელოთ?

1. პერიოდი 1-მდე ოდესმე გქონიათ თქვენ ან ვინმე თქვენს ნაცნობს ჯილეხი?

| რესპოდენტი 1 | რესპოდენტი 2 |
| --- | --- |
| □ დიახ  □ არა  □ არ ვიცი | □ დიახ  □ არა  □ არ ვიცი |

1. პერიოდი 1-მდე ოდესმე თქვენი ან თქვენი ნაცნობის ცხოველი დაცემულა ჯილეხით?

| რესპოდენტი 1 | რესპოდენტი 2 |
| --- | --- |
| □ დიახ  □ არა  □ არ ვიცი | □ დიახ  □ არა  □ არ ვიცი |

1. პერიოდი 1-მდე მიგიღიათ, თუ არა ინფორმაცია ცხოველებში დაავადება ჯილეხის დადგენისა და პრევენციის შესახებ?

| რესპოდენტი 1 | რესპოდენტი 2 |
| --- | --- |
| □ დიახ  □ არა  □ არ ვიცი | □ დიახ  □ არა  □ არ ვიცი |

1. პერიოდი 1-ის შემდეგ მიგიღიათ, თუ არა ინფორმაცია ცხოველებში დაავადება ჯილეხის დადგენისა და პრევენციის შესახებ?

| რესპოდენტი 1 | რესპოდენტი 2 |
| --- | --- |
| □ დიახ  □ არა  □ არ ვიცი | □ დიახ  □ არა  □ არ ვიცი |

53. როგორ გირჩევნიათ მიიღოთ ინფორმაცია ჯილეხის შესახებ? თუ უპირატესობას ანიჭებთ სხვა წყაროს, გთხოვთ, მოგვაწოდოთ გვითხრათ:

| რესპოდენტი 1 | რესპოდენტი 2 |
| --- | --- |
| □ ვეტერინარისაგან ვაქცინაციის კამპანიის ფარგლებში  □ ვეტერინარისაგან, როდესაც ცხოველს ჰქონდა ჯანმრთელობის პრობლემები  □ სოფლის-მეურნეობის საკონსულტაციო ცენტრებიდან  □ ხელმძღვანელებისგან სოფელში  გამართულ შეხვედრებზე  □ რადიოთი, ტელევიზიით  □ სხვა ________________________________ | □ ვეტერინარისაგან ვაქცინაციის კამპანიის ფარგლებში  □ ვეტერინარისაგან, როდესაც ცხოველს ჰქონდა ჯანმრთელობის პრობლემები  □ სოფლის-მეურნეობის საკონსულტაციო ცენტრებიდან  □ ხელმძღვანელებისგან სოფელში  გამართულ შეხვედრებზე  □ რადიოთი, ტელევიზიით  □ სხვა ________________________________ |

54. თქვენი აზრით, რომელ ცხოველს აქვს დაავადება ჯილეხით დაინფიცირების რისკი?

| რესპოდენტი 1 | | | | რესპოდენტი 2 | | | |
| --- | --- | --- | --- | --- | --- | --- | --- |
| დიახ | არ | არ ვიცი |  | დიახ | არ | არ ვიცი |  |
| □  □  □  □  □  □  □ | □  □  □  □  □  □  □ | □  □  □  □  □  □  □ | მსხვილფეხა პირუტყვი  ცხვარი  თხა  ღორი  ცხენი  ძაღლი  სხვა _________________ | □  □  □  □  □  □  □ | □  □  □  □  □  □  □ | □  □  □  □  □  □  □ | მსხვილფეხა პირუტყვი  ცხვარი  თხა  ღორი  ცხენი  ძაღლი  სხვა _________________ |

55. გადადის თუ არა დაავადება ჯილეხი ცხოველიდან ადამიანზე?

| რესპოდენტი 1 | რესპოდენტი 2 |
| --- | --- |
| □ დიახ  □ არა  □ არ ვიცი | □ დიახ  □ არა  □ არ ვიცი |

56. შესაძლოა თუ არა დაავადება ჯილეხის პრევენცია ცხოველებში?

| რესპოდენტი 1 | რესპოდენტი 2 |
| --- | --- |
| □ დიახ  □ არა  □ არ ვიცი | □ დიახ  □ არა  □ არ ვიცი |

57. წარმოადგენს თუ არა პრობლემას დაავადება ჯილეხი თქვენს რეგიონში?

| რესპოდენტი 1 | რესპოდენტი 2 |
| --- | --- |
| □ დიახ  □ არა  □ არ ვიცი | □ დიახ  □ არა  □ არ ვიცი |

57.1. რატომ თვლით რომ დაავადება ჯილეხი არ წარმოადგენს პრობლემას?

| რესპოდენტი 1 | რესპოდენტი 2 |
| --- | --- |
| ______________________________________  ______________________________________ | _______________________________  _______________________________ |

58. უფასო ვაქცინაციის შემთხვევაში, აცრით, თუ არა ჯილეხის წინააღმდეგ თქვენს ცხოველებს?

| რესპოდენტი 1 | რესპოდენტი 2 |
| --- | --- |
| □ დიახ  □ არა  □ არ ვიცი | □ დიახ  □ არა  □ არ ვიცი |

1. აცრით თუ არა თქვენს ცხოველებს ჯილეხის წინააღმდეგ თუ ვაქცინის საფასური თქვენ უნდა გადაიხადოთ?

| რესპოდენტი 1 | რესპოდენტი 2 |
| --- | --- |
| □ დიახ  □ არა  □ არ ვიცი | □ დიახ  □ არა  □ არ ვიცი |

1. რას მოიმოქმედებთ თუ ერთ-ერთი თქვენი ცხოველი უეცრად დაავადდება? [*მონიშნეთ ყველა შესაბამისი პასუხი*]

| რესპოდენტი 1 | რესპოდენტი 2 |
| --- | --- |
| □ ვუმკურნალებ ანტიბიოტიკებით  □ დავკლავ  □ მოვარიდებ სხვა ცხოველებს  □ დავუძახებ კერძო ვეტერინარს  □ დავუძახებ სახელმწიფო ვეტერინარს  □ სხვა ________________________ | □ ვუმკურნალებ ანტიბიოტიკებით  □ დავკლავ  □ მოვარიდებ სხვა ცხოველებს  □ დავუძახებ კერძო ვეტერინარს  □ დავუძახებ სახელმწიფო ვეტერინარს  □ სხვა ________________________ |

1. რას მოიმოქმედებთ თუ ერთ-ერთი თქვენი ცხოველი უეცრად დაეცემა? [*მონიშნეთ ყველა შესაბამისი პასუხი*]

| რესპოდენტი 1 | რესპოდენტი 2 |
| --- | --- |
| □ სხვა ცხოველებს ვუმკურნალებ  ანტიბიოტიკებით  □ გამოვიყენებ ხორცად  □ გავყიდი ლეშს  □ დავმარხავ ლეშს  □ კერძო ვეტერინარს დავუძახებ  □ სახელმწიფო ვეტერინარს გამოვიძახებ  □ სხვა ________________________ | □ სხვა ცხოველებს ვუმკურნალებ  ანტიბიოტიკებით  □ გამოვიყენებ ხორცად  □ გავყიდი ლეშს  □ დავმარხავ ლეშს  □ კერძო ვეტერინარს დავუძახებ  □ სახელმწიფო ვეტერინარს გამოვიძახებ  □ სხვა ________________________ |

1. რომელ ენაზე გირჩევნიან ჯილეხთან დაკავშირებული საგანმანათლებლო მასალის მიღება?

| რესპოდენტი 1 | რესპოდენტი 2 |
| --- | --- |
| □ ქართული  □ რუსული  □ აზერბაიჯანული  □ სომხური  □ სხვა_________________ | □ ქართული  □ რუსული  □ აზერბაიჯანული  □ სომხური  □ სხვა_________________ |

1. რა განათლება გაქვთ მიღებული?

| რესპოდენტი 1 | რესპოდენტი 2 |
| --- | --- |
| □ დაწყებითი  □ საშუალო  □ პროფესიული  □ უმაღლესი | □ დაწყებითი  □ საშუალო  □ პროფესიული  □ უმაღლესი |

ინტერვიუს დროს დამატებული კომენტარი:

___________________________________________________________________________________________

___________________________________________________________________________________________

___________________________________________________________________________________________

გმადლობთ, რომ დაგვითმეთ დრო და მონაწილეობა მიიღეთ ჩვენს კვლევაში. მადლიერების ნიშნად გთხოვთ, მიიღოთ ეს მასალა რომელშიც ნაჩვენებია თქვენთვის, თქვენი ოჯახისათვის და თქვენი ცხოველებისათვის ჯილეხის პრევენციის გზები. გისურვებთ ჯანმრთელობას და წარმატებას!

ინტერვიუს დასრულების დრო: **__ __ : __ __**

მხოლოდ ინტერვიუერებისათვის ინტერვიუს დასრულების შემდგომ შესავსებად

| ადგილმდებარეობა | GPS კოორდინატები |
| --- | --- |
| ცხოველების ღამის სადგომი | 3 ან მეტი სატელიტი? □ დიახ □ არა  განედი (N): _______________ გრძედი (E): __________________  სიმაღლე ზღვის დონიდან (Elevation)____________ მეტრი |

შენიშვნები დამატებითი საძოვრის ისტორიის შესახებ ბოლო 30 დღის განმავლობაში: ______________________________________________________________________________________________________________________________________________________________________________________

იმ შემთხვევაში თუ ერთზე მეტი რესპოდენტი გამოიკითხა, ჩატარდა თუ არა მათი გამოკითხვა ერთდროულად?

□ დიახ □ არა

რა სიხშირით ეთანხმებოდნენ ერთმანეთს რესპოდენტები?

□ ყოველთვის □ ზოგჯერ □ არასოდეს

**ნაწილი 12**. **ცხოველთა კლასიფიკაცია**

შეავსეთ ეს ნაწილი, შემთხვევის სოფელში ინტერვიუს ჩასატარებლად გამგზავრებამდე.

მფლობელის საცხოვრებელი მისამართი: სოფელი/ქალაქი: __________________________

მუნიციპალიტეტი: ________________________

რეგიონი: _________________________________

დაავადების დადგენის თარიღი (რიცხვი/თვე/წელი): __ __ / __ __ / __ __

არ არსებობის შემთხვევაში დაცემის თარიღი (რიცხვი/თვე/წელი): __ __ / __ __ / __ __

ექსპერტიზის ნომერი ______ ექსპერტიზის თარიღი (რიცხვი/თვე/წელი):__ __ / __ __ / __ __

ცხოველის საიდენტიფიკაციო ნომერი (თუ ხელმისაწვდომია): _________________________

საიდენტიფიკაციო ნომრის ტიპი: □ საყურე ნიშანი □ ცხოველის აღწერილობა

□ სხვა, დააკონკრეტეთ ___________

**ინფორმაციის შეგროვების შემდეგ, შეამოწმეთ შემდეგი**

| შეამოწმეთ შევსების შემდეგ | ველზე გასვლამდე შესასრულებელი დავალება |
| --- | --- |
| □ | შემთხვევის საინდენტიფიკაციო ნომერი მოწოდებული იქნა პროექტის კოორდინატორის მიერ  **შემთხვევის** კვლევის კითხვარის საინდენტიფიკაციო ნომერი: __ __ __ __ - __ __ __ __ - __  **სოფელი-კონტროლი** კვლევის კითხვარის საინდენტიფიკაციო ნომერი: __ __ __ __ - __ __ __ __ - __ __ __  **არეალი-კონტროლი** კვლევის კითხვარის საინდენტიფიკაციო ნომერი: __ __ __ __ - __ __ __ __ - __ __ __ |
| □ | დაწერეთ კვლევის კითხვარის საინდენტიფიკაციო ნომერი ზედა ყოველ გვერდიზე |
| □ | დაწერეთ **თარიღი1** (დაავადების დადასტურების თარიღი / არ არსებობის შემთხვევაში დაცემის თარიღი) ზედა ყოველ გვერდზე |
| □ | დარწმუნდით რომ ცხოველის შემთხვევა დადასტურებულია ლაბორატორიულ კვლევებზე დაყრდნობით [*აირჩიეთ ყველა შესაბამისი*]  □ ბიოლოგიური, მიკროსკოპიული  □ ბაქტერიოლოგიური  □ PCR – მოლეკულური |
| □ | მუნიციპალიტეტის ვეტერინარებთან დაკავშირება და გასაუბრებების კოორდინირება |
| □ | შეამოწმეთ რა ენაზე საუბრობენ ცხოველების მეპატრონეები და მწყემსები, რომ განისაზღვროს, რა ენაზე შედგენილი კითხვარი უნდა იქნეს გამოყენებული და არის, თუ არა საჭირო სხვა ინტერვიუ  □ ქართული □ რუსული □ აზერბაიჯანული □სომხური |
| □ | შეირჩა ადგილი - კონტროლის სოფლები:  ადგილი - კონტროლის სოფელი 1 ___________________  ადგილი - კონტროლის სოფელი 2 ___________________ |
| □ | ცხოველის სახეობები: _____________________________________ |
